# White Matter Microstructural Alterations in Type 2 Diabetes: A Combined UK Biobank Study of Diffusion Tensor Imaging and Neurite Orientation Dispersion and Density Imaging

**DOI:** 10.3390/medicina61030455

**Published:** 2025-03-06

**Authors:** Abdulmajeed Alotaibi, Mostafa Alqarras, Anna Podlasek, Abdullah Almanaa, Amjad AlTokhis, Ali Aldhebaib, Bader Aldebasi, Malak Almutairi, Chris R. Tench, Mansour Almanaa, Ali-Reza Mohammadi-Nejad, Cris S. Constantinescu, Rob A. Dineen, Sieun Lee

**Affiliations:** 1 Department of Radiological Sciences, School of Applied Medical Sciences, King Saud bin Abdul-Aziz University for Health Sciences, Riyadh 14611, Saudi Arabia; otaibaibdulm@ksau-hs.edu.sa (A.A.);; 2 Division of Mental Health and Clinical Neurosciences, School of Medicine, University of Nottingham, Nottingham NG7 2RD, UK; 3 Precision Imaging Beacon, University of Nottingham, Nottingham NG7 2RD, UK; 4 King Abdullah International Medical Research Center (KAIMRC), Riyadh 11481, Saudi Arabia; 5 Research and Innovation Unit, College of Applied Medical Sciences, King Saud bin Abdul-Aziz University for Health Sciences, Riyadh 14611, Saudi Arabia; 6 National Institute for Health Research (NIHR) Nottingham Biomedical Research Centre, Queens Medical Centre, Nottingham NG7 2UH, UK; 7 Tayside Innovation MedTech Ecosystem (TIME), University of Dundee, Dundee DD1 4HN, UK; 8 Department of Radiology and Medical Imaging, King Abdulaziz Medical City, National Guard Health Affairs, Riyadh 11426, Saudi Arabia; 9 Department of Radiological Sciences, School of Health and Rehabilitation Sciences, Princess Nourah bint Abdulrahman University, Riyadh 11564, Saudi Arabia; 10 Radiology Department, Armed Forces Hospital, Al-Kharj 16274, Saudi Arabia; 11 Radiological Sciences Department, College of Applied Medical Sciences, King Saud University, Riyadh 11451, Saudi Arabia; 12 Department of Neurology, Cooper Neurological Institute, Cooper Medical School at Rowan University, Camden, NJ 08103, USA

**Keywords:** microstructural imaging, diffusion imaging, type 2 diabetes, white matter, neurite density, orientation dispersion, isotropic volume fraction

## Abstract

*Background and objectives:* Type 2 diabetes mellitus (T2DM) affects brain white matter microstructure. While diffusion tensor imaging (DTI) has been used to study white matter abnormalities in T2DM, it lacks specificity for complex white matter tracts. Neurite orientation dispersion and density imaging (NODDI) offers a more specific approach to characterising white matter microstructures. This study aims to explore white matter alterations in T2DM using both DTI and NODDI and assess their association with disease duration and glycaemic control, as indicated by HbA1c levels. *Methods and Materials*: We analysed white matter microstructure in 48 tracts using data from the UK Biobank, involving 1023 T2DM participants (39% women, mean age 66) and 30,744 non-T2DM controls (53% women, mean age 64). Participants underwent 3.0T multiparametric brain imaging, including T1-weighted and diffusion imaging for DTI and NODDI. We performed region-of-interest analyses on fractional anisotropy (FA), mean diffusivity (MD), axial diffusivity (AD), radial diffusivity (RD), orientation dispersion index (ODI), intracellular volume fraction (ICVF), and isotropic water fraction (IsoVF) to assess white matter abnormalities. *Results:* We observed reduced FA and ICVF, and increased MD, AD, RD, ODI, and IsoVF in T2DM participants compared to controls (*p* < 0.05). These changes were associated with longer disease duration and higher HbA1c levels (0 < r ≤ 0.2, *p* < 0.05). NODDI identified microstructural changes in white matter that were proxies for reduced neurite density and disrupted fibre orientation, correlating with disease progression and poor glucose control. In conclusion, NODDI contributed to DTI in capturing white matter differences in participants with type 2 diabetes, suggesting the feasibility of NODDI in detecting white matter alterations in type 2 diabetes. Type 2 diabetes can cause white matter microstructural abnormalities that have associations with glucose control. *Conclusions:* The NODDI diffusion model allows the characterisation of white matter neuroaxonal pathology in type 2 diabetes, giving biophysical information for understanding the impact of type 2 diabetes on brain microstructure. Future research should focus on the longitudinal tracking of these microstructural changes to better understand their potential as early biomarkers for cognitive decline in T2DM.

## 1. Introduction

Type 2 diabetes mellitus (T2DM) is a common metabolic disorder that impacted approximately 800 million adults in 2022. This represents about 14% of the adult population, with the prevalence rate varying across different regions [1]. For instance, North Africa and the Middle East have a prevalence rate of 9.3%, projected to increase to 16.8% by 2050 [2]. In Latin America and the Caribbean, the prevalence is expected to rise to 11.3% by 2050 [3].

T2DM results in microvascular and macrovascular complications [4], leading to microvascular abnormalities, generally recognised as risk factors for brain atrophy, microstructural alterations, and cognitive impairment [5,6,7]. Using advanced neuroimaging techniques, researchers have recently focused on understanding the link between T2DM and microstructural abnormalities in the brain. Advanced magnetic resonance imaging (MRI) techniques, such as diffusion tensor imaging (DTI), have been widely used to explore microstructural changes in the brain. It has been adopted in several studies to identify global/local network abnormalities and microstructural changes in white matter [8,9,10]. White matter tracts microstructurally affected by T2DM as detected by DTI include the corpus callosum, corona radiata, cingulum, internal/external capsules, fornix, uncinate fasciculus, and corticospinal tracts [5,11,12]. DTI is based on a single-compartment Gaussian diffusion model of brain microstructure with anisotropic water diffusion [13]. Although DTI is sensitive to white matter microstructural changes, it lacks specificity for the tissue microstructure [14,15,16]. When two or more distinct tissues with heterogeneous diffusion properties are present in a single voxel, the interpretation of DTI measures becomes more complicated [16]. Furthermore, DTI may not be an appropriate diffusion model to describe the non-Gaussian diffusion in biological tissues [17].

An alternative biophysical diffusion model, known as neurite orientation dispersion and density imaging (NODDI), has been proposed to theoretically provide more specific characterisations of white matter microstructure [18]. NODDI uses a multi-shell diffusion model with a high angular resolution that describes brain tissue as a simplified combination of three compartments and separates the signals from each compartment [18]. A Watson distribution assumption of sticks models the first compartment, known as the intracellular or intra-neurite compartment (axons and dendrites), and the signal of each stick is assumed to be a perfectly anisotropic diffusion tensor with a perpendicular diffusivity equal to zero. Additionally, it represents extracellular space with a Gaussian anisotropic diffusion and a free water compartment that represents an isotropic Gaussian diffusion, such as cerebrospinal fluid (CSF) [18]. NODDI uses three scalar parameters, as follows: neurite density index (NDI), also known as intra-cellular volume fraction (ICVF); orientation dispersion index (ODI) (0 for perfectly aligned straight fibres and 1 for entirely isotropic); and free water fraction or isotropic volume fraction (IsoVF) [19]. These parameters estimate the orientation dispersion and neurite density, contributing to conventional DTI parameters such as FA [18].

Although NODDI has not been widely applied in diabetes, it is promising for evaluating the complexity of white matter neuroaxonal pathology. A previous study has demonstrated that NODDI parameters can detect differences between T2DM and non-T2DM participants, suggesting their promising role as a biomarker for assessing white matter microstructural deficits in T2DM patients [20]. However, this study was performed on 18 patients with T2DM only, the slice thickness was large, and the diffusion encoding direction of 25 was relatively small to estimate the fibre orientation. These technical and sample size limitations may have limited the quantification of NODDI-derived parameters in T2DM patients.

Herein, the brain white matter microstructural changes in a large cohort of participants with T2DM from a population-level study (the UK Biobank [21,22]) using measures derived from DTI and NODDI were explored. The aims of this large-scale study are (1) to investigate brain white matter microstructural alterations of T2DM participants using NODDI and DTI to determine their use in detecting global and regional white matter differences; and (2) to examine the association between the altered NODDI and DTI metrics, the duration of diabetes, and the glycated haemoglobin (HbA1c) levels.

## 2. Materials and Methods

### 2.1. UK Biobank Cohort

UK Biobank is a population-level study of 500,000 people aged 40 to 69 years old recruited between 2006 and 2010, with data collected in 22 centres across England, Scotland, and Wales [23]. All participants provided informed consent forms. Ethical approval was obtained from the Northwest Multi-Centre Research Ethics Committee. The current study was approved under the UK Biobank application ID 43822. Details of the UK Biobank cohort and consent are available on the UK Biobank website [23].

### 2.2. Clinical Characteristics and Sample Selection

A total of 31,767 participants were included in this study (1023 participants with T2DM and 30744 non-T2DM participants). Touch-screen questionnaires, health exams, brief computer-assisted interviews, biological samples, and imaging data were among the baseline assessments [22]. At the time of the imaging visit, data on age, sex, qualification, diabetes history, body mass index (BMI), blood pressure (BP), glycated haemoglobin (HbA1c), cardiovascular disease history, and neurological disorders were collected and reported. Socioeconomic status, family history, lifestyle, and health status were among the datasets acquired through questionnaires and interviews. Participants with T2DM were included in the study based on self-reports and hospital inpatient ICD10 (International Classification of Diseases) records across all UK hospitals. To ensure the specificity of the sample, only individuals with T2DM who did not have any neurological diseases were selected. Neurological conditions such as stroke, haemorrhage, cerebral infarction, Parkinson’s disease, Alzheimer’s disease, epilepsy, head injuries, or tumours were considered exclusion criteria. Additionally, participants with other neurological disorders were excluded if they reported such conditions through touch-screen questionnaires or verbal interviews. This strict selection ensures that the study focuses on individuals with T2DM, excluding any confounding effects from neurological disorders. Furthermore, participants with a BMI exceeding 33 were excluded from the study to control for obesity-related confounding factors. For comparison, UK Biobank participants who had no history of diabetes or neurological disorders were included as the control group. This stringent approach allowed for the comparison of T2DM-related brain changes with a healthy, non-diabetic cohort.

### 2.3. Brain MRI Acquisition

Participants in UK Biobank were invited to attend MRI scans at one of three dedicated imaging centres equipped with identical scanners (3T Siemens Skyra, software VD13A (https://www.siemens-healthineers.com/digital-health-solutions/syngovia-view-go, accessed on 28 December 2024) via Siemens Healthcare) and using a Siemens 32-channel receive head coil. Diffusion images were acquired using echo-planar imaging with a single-shot Stejskal–Tanner pulse sequence in 100 diffusion-weighted directions (b-values = 0, 1000, and 2000 s/mm^2^). T1-weighted structural brain images were acquired using a three-dimensional MPRAGE sequence. The brain imaging protocol details are available online and in the UK Biobank imaging report [24,25].

### 2.4. Diffusion Image Processing

In the diffusion data, the anterior–posterior (AP) encoding direction was corrected for eddy currents, head motion, and outlier slices (individual slices in the 4D data). The diffusion images were corrected using the eddy tool in the first step of this pipeline [25,26,27]. This step required knowledge of the “best” b = 0 images in the AP direction. The gradient distortion correction was applied after eddy to produce a more precise correction [28]. The diffusion images were analysed based on tract-skeleton (TBSS) processing. Then, the output was a wide range of diffusion MRI-derived measures within different tract areas, including (1) measurements derived from diffusion-tensor modelling and (2) measurements derived from microstructural model fitting. More details on image processing are available in previous UK Biobank reports [21,25,29].

#### 2.4.1. DTI and NODDI Fitting

The DTI fitting was applied to the b = 1000 shell (50 directions) using the DTIFIT tool [30]. In addition to DTI fitting, the entire two-shell diffusion dataset was fed into NODDI modelling, performed with the AMICO (Accelerated Microstructure Imaging via Convex Optimization) tool [31]. This aimed to generate voxel-wise microstructural parameters, such as ICVF, ODI, and IsoVF.

#### 2.4.2. White Matter Tract Skeleton Analysis

The DTI FA image was fed into TBSS [32], which aligned the FA image onto a standard-space-white-matter skeleton, with additional alignment that improved the original TBSS skeleton projection by employing a high-dimensional FNIRT-based warping technique [33]. The resulting standard-space warp was applied to DTI/NODDI output maps. For each DTI/NODDI map, the skeletonised images were averaged within a set of 48 standard-space tract masks defined by the Johns Hopkins University group (JHU-ICBM atlas) [34] to generate a set of diffusion imaging-derived phenotypes (IDPs).

### 2.5. Modelling in UK Biobank Brain Imaging

A huge imaging dataset, such as the UK Biobank data, may contain confounding factors that must be corrected before running the final statistical analysis. In this study, the following imaging and demographic features were de-confounded from the brain IDPs: age, sex, head motion, head size scaling, bed/table position in the scanner (for the three axes X, Y, Z), acquisition date/time, and imaging sites [21,35]. A linear model was concurrently used (i.e., in a single regression-based de-confounding model employing all variables combined). For instance, Y is the N-vector of interest, and X is the N-by-P matrix of all variables. In that case, the confound-adjusted variables are the residuals from the linear model fit Y to X [35].

### 2.6. Statistical Analysis

Statistical analysis was performed using SPSS 27.0 (IBM, New York, NY, USA). Continuous data were presented as mean ± standard deviation, and categorical data were presented as percentages. Intergroup differences, including clinical characteristics and brain white matter differences, were compared by Student’s *t*-test and Pearson’s chi-square test. Pearson correlation analyses were conducted to explore the association between HbA1c, disease duration, and diffusion measures. Pearson’s correlation coefficient was considered weak (0 < r ≤ 0.4). The defined *p*-value threshold was set to a *p*-value of 0.05 (false discovery rate).

## 3. Results

### 3.1. Clinical Characteristics

**Figure 1:**
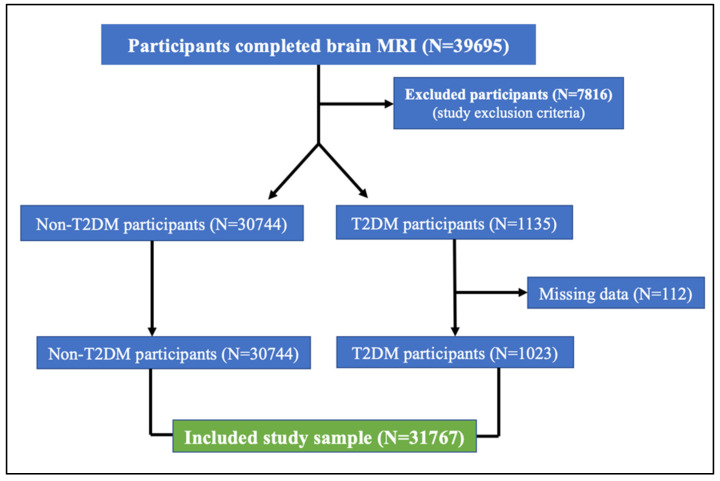
Flowchart of the included study sample based on the study inclusion/exclusion criteria.

In this UK Biobank study, 1023 participants with T2DM (39% women, mean age 66 years) and 30,744 non-T2DM participants (53% women, mean age 64 years) had undergone diffusion MRI (Figure 1). They were retained in this analysis after applying the inclusion/exclusion criteria. The clinical characteristics of both groups are presented in Table 1.

### 3.2. White Matter Intergroup Differences and Association with Metabolic Profile: Overview

Parametric maps of DTI and NODDI were generated for each subject. This large sample study revealed subtle but global white matter microstructural changes in the participants with T2DM compared to non-T2DM individuals, which were reflected by reduced FA and ICVF and increased MD, AD, RD, ODI, and IsoVF. These subtle alterations had weak correlations with disease duration and HbA1c.

#### 3.2.1. DTI Intergroup White Matter Differences

**Figure 2:**
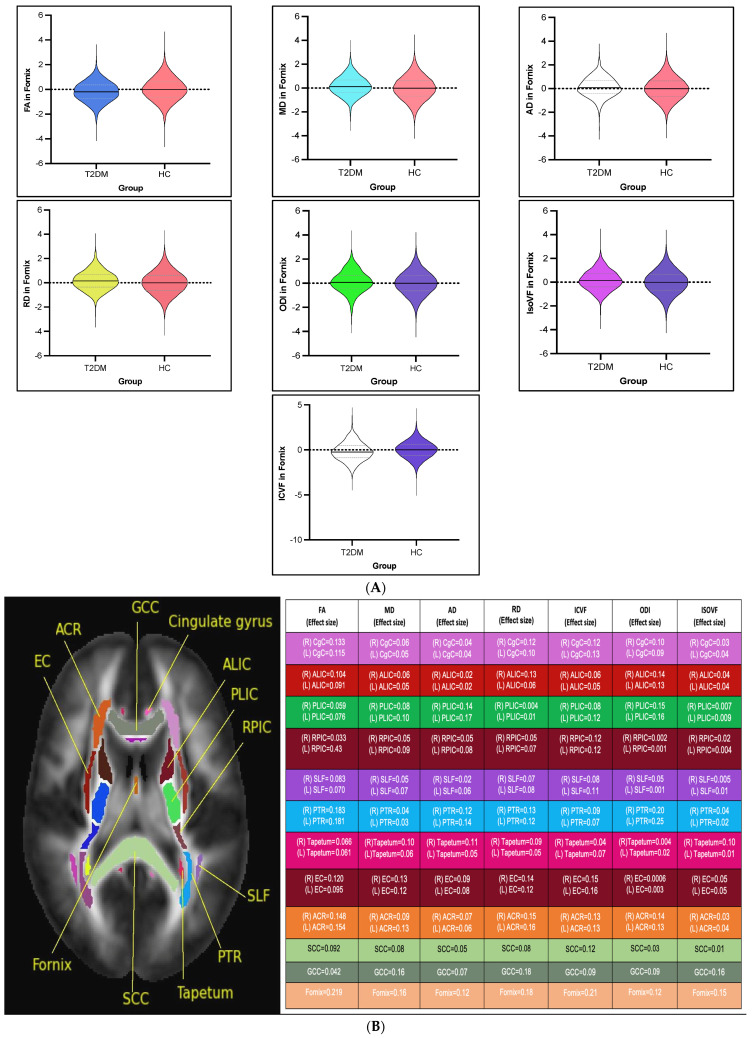
(**A**) Violin plots for the fornix as a selected white matter tract (from the de-confounded dataset) with a larger effect size to visualise the intergroup DTI and NODDI-based white matter alterations in patients with T2DM (*p* < 0.05, false discovery rate adjustment). (**B**) Global alterations with the effect sizes of each measure in each tract over the whole brain. Genu of corpus callosum (GCC), fornix, cingulate of gyrus, superior longitudinal fasciculus (SLF), anterior corona radiata (ACR), anterior limb of the internal capsule (ALIC), posterior limb of the internal capsule (PLIC), posterior thalamic radiation (PTR), tapetum, splenium of corpus callosum (SCC), external capsule (EC), and retro-lenticular part of the internal capsule (RPIC).

FA showed a significant reduction in most of the examined white matter tracts of participants with T2DM compared to non-T2DM individuals (all *p* < 0.05, d ≤ 0.3). Moreover, MD, AD, and RD showed a significant increase in the majority of the investigated white matter tracts of participants with T2DM (all *p* < 0.05, d ≤ 0.3) (see Figure 2A,B). Details on statistics of DTI differences are shown in Supplementary Table S1A–D.

#### 3.2.2. NODDI Intergroup White Matter Differences

ICVF was significantly reduced in the majority of the investigated white matter tracts of participants with T2DM compared to non-T2DM individuals (all *p* < 0.05, d < 0.3). ODI and IsoVF were significantly increased in the majority of investigated white matter tracts of participants with T2DM compared to non-T2DM individuals (all *p* < 0.05, d < 0.3). In contrast, there was a decrease in ODI in the bilateral posterior limbs of the internal capsules, bilateral superior corona radiata, and right posterior corona radiata (*p* < 0.05, d < 0.3) (see Figure 2A,B). Details on statistics of NODDI differences are shown in Supplementary Table S2A–D.

### 3.3. Diffusion Measures and Correlation with Metabolic Profile

#### 3.3.1. Disease Duration, HbA1c, and DTI

**Figure 3:**
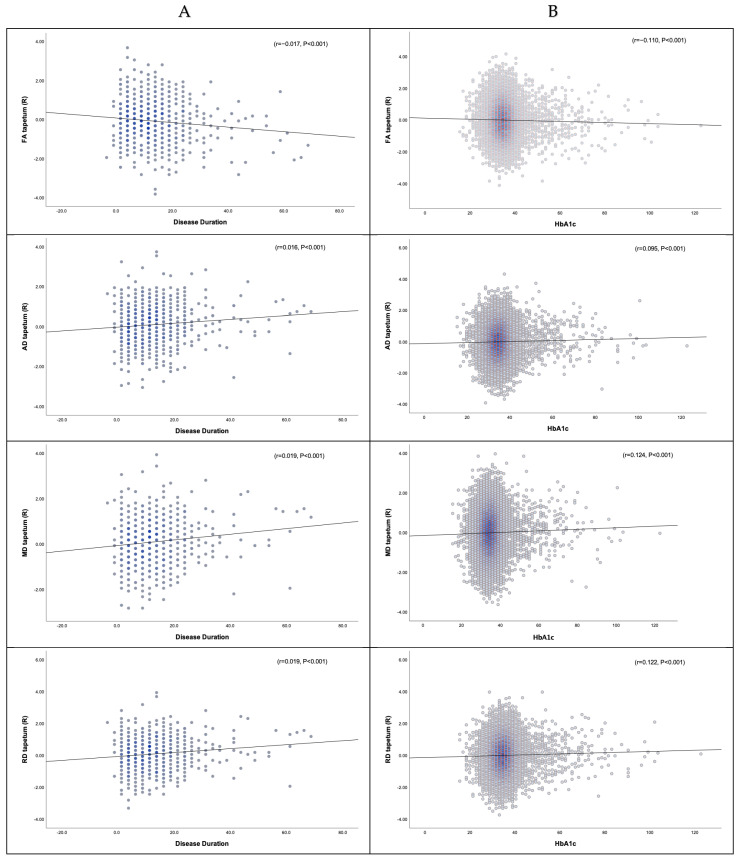

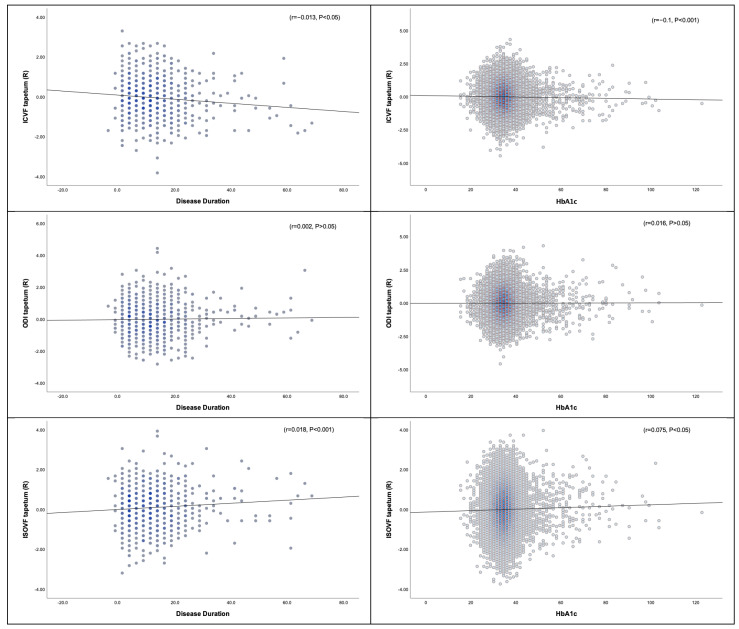
The right tapetum is a selected white matter tract from the de-confounded dataset to visualise the association between the white matter change detected by DTI/NODDI and the metabolic profile. (**A**) Association between white matter alterations in the right tapetum and disease duration; (**B**) Association between white matter alterations in the right tapetum and HbA1c.

Correlation analysis showed a weak association between disease duration, HbA1c, and DTI changes in white matter tracts of participants with T2DM. Reduced FA and increased MD, AD, and RD correlated with longer disease duration in most examined white matter tracts (all *p* < 0.05) (0 < r < 0.2). Similarly, reduced FA and increased MD, AD, and RD weakly correlated with higher levels of HbA1c (all *p* < 0.05) (0 < r < 0.2) (see Figure 3, Table 2, and Supplementary Table S3).

#### 3.3.2. Disease Duration, HbA1c, and NODDI

**Figure 4:**
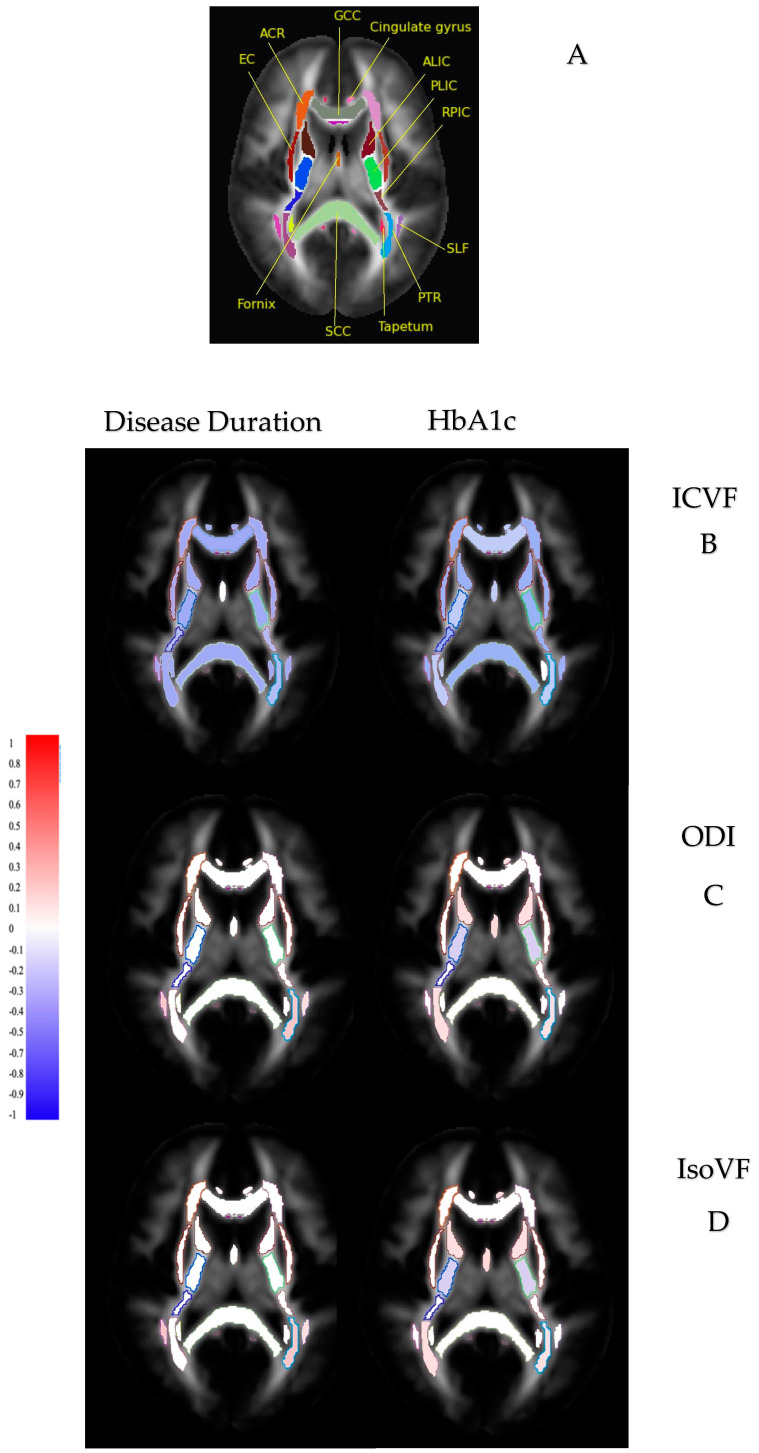
Correlations between disease duration and HbA1c with major white matter tracts in participants with T2DM. White matter structures were selected only for visualisation purposes. (**A**) White matter tracts included. (**B**) Altered ICVF and disease duration/HbA1c. (**C**) Altered ODI and disease duration/HbA1c. (**D**) Altered IsoVF and disease duration/HbA1c. Red: positive correlation; blue: negative correlation. These illustrated correlations are based on a brain model derived from a white matter atlas.

Reduced ICVF and increased ODI and IsoVF had weak correlations with longer disease duration and HbA1c in most examined white matter tracts (all *p* < 0.05) (0 < r < 0.2). Likewise, reduced ICVF and increased ODI and IsoVF had a weak but significant correlation with higher levels of HbA1c (all *p* < 0.05) (0 < r < 0.2) (see Figure 3 and Figure 4, Table 2, and Supplementary Table S3).

## 4. Discussion

Using these large-scale diffusion imaging data from 31,767 participants from the UK Biobank, the ability of DTI and NODDI-derived metrics to capture microstructural changes in the white matter in participants with T2DM was evaluated. Moreover, we examined whether disease duration and HbA1c were associated with altered DTI/NODDI parameters.

In T2DM participants, white matter tracts and fibres mostly show reduced FA, ICVF, and increased MD, AD, RD, ODI, and IsoVF, as presented in Supplementary Table S4. Reduced anisotropy and increased diffusivity, reflected by reduced FA and higher MD, confirm widespread microstructural alterations and broadly agree with previous DTI studies (see the previous reviews [5,11,12]). The elevation of AD and RD in participants with T2DM might lead to changes in FA and MD, as AD is linked to axonal integrity and RD is linked to myelin abnormalities. Therefore, the findings of the UK Biobank study suggest that increased interstitial space between the myelin-covered axons (expansion of extracellular space due to the degeneration of fibres/axonal injury) and reduced myelin integrity play a role in white matter deficits in T2DM participants [15,36,37,38]. A recent study showed that T2DM patients had increased MD, AD, and RD values in association fibres, aligning with our findings. The MD increase indicated a reduction in cell density and potential axonal loss, while the increased RD reflected the disruption of the myelin sheath. AD changes were more subtle but suggested axonal degeneration, particularly in the posterior portion of the superior longitudinal fasciculus (SLF). Despite the extensive changes in MD, AD, and RD, no significant differences were found in FA values between T2DM patients and HCs [39]. Another study revealed a significant correlation between microstructural abnormalities in white matter and cognitive impairment, highlighting the potential of advanced diffusion techniques as a tool for assessing neural changes in this population [40].

The reduction in ICVF in the white matter of T2DM in this study was consistent with a previous study [20], which indicates reduced neurite density. The reduced ICVF or neurite density in T2DM is likely relevant to structural changes, such as axonal loss, axonal damage, or axonal demyelination/degeneration [41]. Furthermore, the decreased neurite density in the participants with T2DM may be accompanied by axonal disorganisation, myelin loss, or loss of fibre orientation coherence that may lead to increased ODI in response to the alterations in white matter [11,41,42,43,44]. Interestingly, decreased ODI is observed in bilateral posterior limbs of internal capsules, bilateral superior corona radiata, and right posterior corona radiata, which may reflect the loss of neuronal/crossing fibres, reorganisation of fibres, or reduced axonal dispersion in T2DM [41,45,46]. In addition, higher coherence of axonal packing in the white matter of T2DM patients, reflected by the decrease in ODI, may suggest either a reduced collateral branching driven by the pathology or alterations in the morphology of an individual axon. Lastly, the elevated IsoVF may indicate vasogenic oedema or increased extracellular free water, which may be caused by the disruption of BBB due to T2DM [47] or may indicate a degree of atrophy by reflecting cell shrinkage and decreased tissue volume fraction [48].

By examining the relationship between the metabolic profile and white matter intergroup differences, statistically significant associations were identified between altered DTI and NODDI parameters in T2DM participants and glycaemic control/disease duration. The neurite density in the white matter of T2DM participants, reflected by a reduced ICVF, has a consistent association with disease duration and poorly controlled blood glucose, suggesting that T2DM participants with longer disease duration and higher plasma glucose tend to have reduced neurite density. Poorly controlled blood glucose and disease duration correlate with loss of coherence of white matter fibres or axonal disorganisation, as reflected by higher ODI. However, the ODI association with the metabolic profile was not as consistent as the ICVF correlation, particularly with disease duration. Furthermore, reduced ODI in the posterior limbs of the internal capsule, right posterior corona radiata, and bilateral superior corona radiata shows an association with disease duration, indicating that the loss of neuronal fibres or neurodegeneration may occur with longer disease duration. Finally, the increased interstitial fluids, reflected by increased IsoVF in T2DM participants, were associated with longer disease duration and higher HbA1c levels. However, this correlation has less consistency compared to ICVF. The correlation coefficient is weak, but there is still a statistical relationship, suggesting that the effect of glycaemic control on brain white matter is not major but does have an effect. For this reason, T2DM patients must aim for good glycaemic control to minimise the microstructural impact of T2DM on brain white matter. It is also worth noting that two models with and without age adjustment were tested. The results suggest that age may not affect the association between white matter alterations in T2DM participants and disease duration.

The findings of this study demonstrate that DTI still has potential value as a clinical biomarker in T2DM. Additionally, it shows that the changes captured by NODDI parameters may contribute to FA alterations (especially in areas with complex microstructure [49]) in the brain white matter of T2DM participants. However, NODDI may suffer the constant diffusivity assumption when pathology changes diffusivity. The NODDI model relies on assumptions to speculate the microstructural alterations; nevertheless, the NODDI parameters with different neurological disorders must be interpreted carefully [50,51,52].

The results show that NODDI parameters could detect white matter neuropathological changes in white matter microstructure, suggesting its potential role for monitoring microstructural alterations in T2DM. This study has limitations. First, it is retrospective and based on a biobank rather than a prospective case-control study. Second, there could have been other non-excluded systemic conditions that could impact the DTI and NODDI metrics, such as previous chemotherapy, cardiac surgery, chronic diseases, and psychiatric disorders. Third, further work to improve this novel study is suggested, particularly because the impact of T2DM on brain functions and neurocognition and their association with the altered NODDI parameters was not explored. Moreover, the study did not include a follow-up assessment to monitor the white matter changes longitudinally in T2DM participants. Fourth, T2DM duration was calculated by subtracting the earliest diagnosis age from the assessment-centre-visit age. Furthermore, the diabetes duration in the UK Biobank datasets may not have been accurately completed, and T2DM may have existed before patients were formally diagnosed. Fifth, we could not relate/associate the diffusion metrics with sex interactions and cholesterol due to our UK Biobank limited access. Lastly, the microstructural impact of T2DM on the grey matter was not evaluated in this study.

In summary, the most relevant findings of this comprehensive study are a reduction in FA and NODDI abnormalities in most white matter tracts in T2DM. These alterations show some correlation with disease duration and glucose control. These alterations are summarised in Figure 2B, where the effect size for each tract for each diffusion measurement is mapped, and a representative example is provided for the fornix in Figure 2A. Future research should focus on the longitudinal tracking of these microstructural changes to better understand their potential as early biomarkers for cognitive and microstructural decline in T2DM.

## 5. Conclusions

White matter microstructural abnormalities occur in T2DM participants and are weakly associated with longer disease duration and higher glycated haemoglobin, reflecting poorer glycaemic control. As an advanced diffusion model, NODDI can characterise white matter neuroaxonal pathology in T2DM, giving quantitative-biophysical information for understanding the impact of T2DM on white matter microstructure.

## Figures and Tables

**Table 1:** Clinical characteristics of the included participants from the UK Biobank database.

Cohort Characteristics						
	(*N* = 1023) T2DM (Mean ± SD)%	(*N* = 30744) Non-T2DM (mean ± SD)%	χ^2^/T	Mean Difference	95% CI	
					Lower	Upper
Gender F (%)	339/39%	16,588/53%	172.4	--	--	--
Age (years)	66 ± 7.04	64 ± 7.5	11.2	2.5	2.09	2.9
Systolic blood pressure (mmHg)	142.6 ± 18.05	136.03 ± 18.6	11.3	6.7	5.6	7.9
Diastolic blood pressure (mmHg)	83.7 ± 10.4	81.2 ± 10.4	7.6	2.6	1.9	3.4
Hb1AC (mmol/mol)	47.08 ± 12.06	34.3 ± 3.5	32.6	12.9	12.1	13.6
BMI (kg/m^2^)	29.8 ± 5.1	26.3 ± 4.3	21.3	3.5	3.2	3.8
Disease duration (years)	12.3 ± 9.3	--	--	--	--	--
Education level	Missing 128	Missing 2062				
(A)	183 (17.8%)	4429 (14.4%)	82.03			
(B)	200 (19.5%)	5394 (17.5%)				
(C)	117 (11.4%)	3735 (12.1%)				
(D)	395 (38.6%)	15124 (49.1%)				

±: mean and standard deviation; A: college or university degree; B: A levels/AS levels or equivalent; C: O levels/GCSEs or equivalent; D: CSEs or equivalent; and --: not applicable.

**Table 2:** Number of white matter tracts correlated with disease duration and HbA1c levels, direction, and strength of the correlations (*p* <0.05 after false discovery rate adjustment). WM: white matter.

Diffusion Indices	Disease Duration	HbA1c
Reduced FA	Number of tracts: 31/48 WM tracts	35/48 WM tracts
	Direction: negative correlation	Negative correlation
	Strength: weak (0 < r ≤ 0.2)	Weak (0 < r ≤ 0.2)
Increased MD	Number of tracts: 30/48 WM tracts	35/48 WM tracts
	Direction: positive correlation	Positive correlation
	Strength: weak (0 < r ≤ 0.2)	Weak (0 < r ≤ 0.2)
Increased AD	Number of tracts: 13/48 WM tracts	18/48 WM tracts
	Direction: positive correlation	Positive correlation
	Strength: weak (0 < r ≤ 0.2)	Weak (0 < r ≤ 0.2)
Increased RD	Number of tracts: 39/48 WM tracts	40/48 WM tracts
	Direction: positive correlation	Positive correlation
	Strength: weak (0 < r ≤ 0.2)	Weak (0 < r ≤ 0.2)
Reduced ICVF	Number of tracts: 43/48 WM tracts	40/48 WM tracts
	Direction: negative correlation	Negative correlation
	Strength: weak (0 < r ≤ 0.2)	Weak (0 < r ≤ 0.2)
Increased ODI	Number of tracts: 3/48 WM tracts	23/48 WM tracts
	Direction: positive correlation	Positive correlation
	Strength: weak (0 < r ≤ 0.2)	Weak (0 < r ≤ 0.2)
Increased IsoVF	Number of tracts: 8/48 WM tracts	13/48 WM tracts
	Direction: positive correlation	Positive correlation
	Strength: weak (0 < r ≤ 0.2)	Weak (0 < r ≤ 0.2)

FA: fractional anisotropy; MD: mean diffusivity; AD: axial diffusivity; RD: radial diffusivity; ICVF: intracellular volume fraction; ODI: orientation dispersion index; and IsoVF: isotropic water fraction.

## Data Availability

The data that support the findings of this study are available from the corresponding author upon reasonable request.

## Supplementary Materials

The following supporting information can be downloaded at: https://www.mdpi.com/article/10.3390/medicina61030455/s1, Table S1A: FA and Intergroup Microstructural Differences, Table S1B: MD and Intergroup Microstructural Differences, Table S1C: AD and Intergroup Microstructural Differences, Table S1D: RD and Intergroup Microstructural Differences. Table S2A: ICVF and Intergroup Microstructural Differences, Table S2B: ODI and Intergroup Microstructural Differences, Table S2C: IsoVF and Intergroup Microstructural Differences, Table S3: Associations between disease duration/HbA1c and White Matter changes in T2DM participants. NS = not significant.
