# Supplementary material for: White Matter Microstructural Alterations in Type 2 Diabetes: A Combined UK Biobank Study of Diffusion Tensor Imaging and Neurite Orientation Dispersion and Density Imaging
Source: Medicina (Kaunas). 2025 Mar 6;61(3):455. doi: 10.3390/medicina61030455 (PMC11943854; doi:10.3390/medicina61030455)
Supplement: Supplementary file 1 [file medicina-61-00455-s001.zip › medicina-3425917-supplementary.pdf]

Table S1A. FA and Intergroup Microstructural Differences.

FA WM Tract and Group		N	Mean	Std. Deviation	Mean Difference (95% CI)	P-value	Effect Size
Middle cerebellar peduncle	Non T2DM	30744	0.005450	0.938413	0.01 (-0.04, 0.07)	0.6	.015
	T2DM	1023	-0.008766	0.930215			
Pontine crossing	Non T2DM	30744	0.014516	0.908788	0.03 (-0.01, 0.09)	0.2	.042
	T2DM	1023	-0.023632	0.967857			
Genu of corpus callosum	Non T2DM	30744	0.015710	0.939996	0.17 (0.11, 0.23)	<0.001	.181
	T2DM	1023	-0.154953	0.968473			
Body of corpus callosum	Non T2DM	30744	0.014099	0.975441	0.20 (0.14, 0.26)	<0.001	.208
	T2DM	1023	-0.188581	0.972339			
Splenium of corpus callosum	Non T2DM	30744	0.015518	0.827389	0.07 (0.01, 0.13)	0.01	.092
	T2DM	1023	-0.060867	0.999620			
Fornix	Non T2DM	30744	0.009745	0.933687	0.20 (0.15, 0.25)	<0.001	.219
	T2DM	1023	-0.193458	0.798754			
Corticospinal tract (R)	Non T2DM	30744	0.009919	0.946782	0.03 (-0.02, 0.08)	0.2	.034
	T2DM	1023	-0.022036	0.903851			
Corticospinal tract (L)	Non T2DM	30744	0.011902	0.925655	0.04 (-0.01, 0.10)	0.1	.046
	T2DM	1023	-0.030883	0.960727			
Medial lemniscus (R)	Non T2DM	30744	0.012880	0.929472	0.13 (0.07, 0.19)	<0.001	.142
	T2DM	1023	-0.119164	0.941373			
Medial lemniscus (L)	Non T2DM	30744	0.006319	0.905491	0.14 (0.08, 0.2)	<0.001	.161
	T2DM	1023	-0.139946	0.932239			
Inferior cerebellar peduncle (R)	Non T2DM	30744	0.006266	0.898204	0.08 (0.02, 0.13)	<0.001	.092
	T2DM	1023	-0.076112	0.916704			
Inferior cerebellar peduncle (L)	Non T2DM	30744	0.017283	0.924883	0.11 (0.06, 0.17)	<0.001	.126
	T2DM	1023	-0.099363	0.887749			
Superior cerebellar peduncle (R)	Non T2DM	30744	0.021203	0.928668	0.27 (0.21, 0.33)	<0.001	.296
	T2DM	1023	-0.253829	0.977185			
Superior cerebellar peduncle (L)	Non T2DM	30744	0.015061	0.908900	0.29 (0.23, 0.35)	<0.001	.329
	T2DM	1023	-0.276923	0.957662			
Cerebral peduncle (R)	Non T2DM	30744	0.016233	0.903907	0.13 (0.07, 0.19)	<0.001	.152
	T2DM	1023	-0.121022	0.922485			
Cerebral peduncle (L)	Non T2DM	30744	0.010117	0.946702	0.12 (0.06, 0.18)	<0.001	.130
	T2DM	1023	-0.112698	0.921053			
Anterior limb of internal capsule (R)	Non T2DM	30744	0.011844	0.941595	0.09 (0.03, 0.15)	0.002	.104
	T2DM	1023	-0.086114	0.982984			
Anterior limb of internal capsule (L)	Non T2DM	30744	0.002735	0.952270	0.08 (0.02, 0.14)	0.006	.091
	T2DM	1023	-0.084332	0.992505			
Posterior limb of internal capsule (R)	Non T2DM	30744	-0.000490	0.956689	-0.05 (-0.11, 0.005)	0.7	.059
	T2DM	1023	0.056183	0.997769			
Posterior limb of internal capsule (L)	Non T2DM	30744	0.008421	0.961241	-0.07 (-0.13, -0.013)	0.17	.076
	T2DM	1023	0.081733	0.958225			
	Non T2DM	30744	0.008905	0.957573	0.03 (-0.03, 0.09)	0.3	.033

Retrolenticular part of internal capsule (R)	T2DM	1023	-0.022920	1.017518			
Retrolenticular part of internal capsule (L)	Non T2DM	30744	0.015200	0.907125	0.03 (-0.02, 0.10)	0.2	0.43
	T2DM	1023	-0.024234	1.006436			
Anterior corona radiata (R)	Non T2DM	30744	0.016704	0.883404	0.13 (0.07, 0.19)	<0.001	.148
	T2DM	1023	-0.114314	0.950761			
Anterior corona radiata (L)	Non T2DM	30744	0.009453	0.949038	0.14 (0.08, 0.20)	<0.001	.154
	T2DM	1023	-0.136826	0.925499			
Superior corona radiata (R)	Non T2DM	30744	0.011538	0.947563	0.03 (-0.03, 0.09)	0.3	.034
	T2DM	1023	-0.020285	1.017567			
Superior corona radiata (L)	Non T2DM	30744	0.006058	0.978610	0.03 (-0.03, 0.10)	0.2	.035
	T2DM	1023	-0.028740	1.046162			
Posterior corona radiata (R)	Non T2DM	30744	0.007537	0.966078	0.011 (-0.05, 0.07)	0.7	.012
	T2DM	1023	-0.003977	1.065942			
Posterior corona radiata (L)	Non T2DM	30744	0.015898	0.915362	0.04 (-0.02, 0.10)	0.1	.047
	T2DM	1023	-0.026994	1.030606			
Posterior thalamic radiation (R)	Non T2DM	30744	0.015208	0.920599	0.16 (0.10, 0.22)	<0.001	.183
	T2DM	1023	-0.153359	0.952977			
Posterior thalamic radiata (L)	Non T2DM	30744	0.013604	0.962017	0.17 (0.11, 0.23)	<0.001	.181
	T2DM	1023	-0.160946	0.955437			
Sagittal stratum (R)	Non T2DM	30744	0.015088	0.955336	0.15 (0.09, 0.21)	<0.001	.164
	T2DM	1023	-0.141242	0.960421			
Sagittal stratum (L)	Non T2DM	30744	0.014776	0.906346	0.18 (0.12, 0.24)	<0.001	.204
	T2DM	1023	-0.170771	0.952318			
External capsule (R)	Non T2DM	30744	0.012537	0.902945	0.10 (0.04, 0.16)	<0.001	.120
	T2DM	1023	-0.095713	0.933989			
External capsule (L)	Non T2DM	30744	0.013269	0.948382	0.09 (0.02, 0.15)	<0.001	.095
	T2DM	1023	-0.076815	0.975428			
Cingulum cingulate gyrus (R)	Non T2DM	30744	0.013343	0.937644	0.12 (0.06, 0.18)	<0.001	.133
	T2DM	1023	-0.111779	1.013694			
Cingulum cingulate gyrus (L)	Non T2DM	30744	0.000232	0.949976	0.10 (0.04, 0.17)	0.14	.115
	T2DM	1023	-0.109085	0.981397			
Cingulum hippocampus (R)	Non T2DM	30744	0.000832	0.950110	-0.03 (-0.09, 0.02)	0.2	.041
	T2DM	1023	0.039580	0.956787			
Cingulum hippocampus (L)	Non T2DM	30744	0.013900	0.848218	0.005 (-0.05, 0.06)	0.8	.006
	T2DM	1023	0.008530	0.935880			
Fornix cres+stria terminalis (R)	Non T2DM	30744	0.014593	0.840709	0.16 (0.10, 0.20)	<0.001	.194
	T2DM	1023	-0.148793	0.883083			
Fornix cres+stria terminalis (L)	Non T2DM	30744	0.012292	0.965105	0.14 (0.09, 0.20)	<0.001	.156
	T2DM	1023	-0.137504	0.895269			
Superior longitudinal fasciculus (R)	Non T2DM	30744	0.012050	0.962038	0.07 (0.014, 0.14)	0.01	.083
	T2DM	1023	-0.067619	1.047019			
Superior longitudinal fasciculus (L)	Non T2DM	30744	0.014155	0.930290	0.06 (0.001, 0.12)	0.04	.070
	T2DM	1023	-0.051120	1.017221			
Superior fronto-occipital fasciculus (R)	Non T2DM	30744	0.015462	0.918298	0.19 (0.13, 0.25)	<0.001	.215
	T2DM	1023	-0.182441	0.973257			

Superior fronto-occipital fasciculus (L)	Non T2DM	30744	0.011504	0.973226	0.19 (0.13, 0.25)	<0.001	.200
	T2DM	1023	-0.183004	0.966043			
Uncinate fasciculus (R)	Non T2DM	30744	0.010246	0.976528	0.06 (0.001, 0.12)	0.04	.064
	T2DM	1023	-0.052098	0.974881			
Uncinate fasciculus (L)	Non T2DM	30744	0.008272	0.935015	0.06 (0.005, 0.12)	0.03	.070
	T2DM	1023	-0.057296	0.965086			
Tapetum (R)	Non T2DM	30744	0.006080	0.960705	0.06 (0.001, 0.12)	0.04	.066
	T2DM	1023	-0.056955	0.988803			
Tapetum (L)	Non T2DM	30744	-0.005351	0.878749	0.05 (-0.009, 0.11)	0.5	.061
	T2DM	1023	-0.058803	1.001205			

Table S1B. MD and Intergroup Microstructural Differences.

**MD WM Tracts and Group**		**N**	**Mean**	**Std. Deviation**	**Mean Difference (95% CI)**	**P-value**	**Effect Size**
Middle cerebellar peduncle	Non T2DM	30744	-0.001583	0.945830	0.05 (0.005, 0.10)	0.07	0.05
	T2DM	1023	0.049991	0.904701			
Pontine crossing tract	Non T2DM	30744	-0.010952	0.852366	0.03 (0.02, 0.08)	0.28	0.03
	T2DM	1023	-0.042247	0.923266			
Genu of corpus callosum	Non T2DM	30744	-0.010638	0.869489	0.14 (0.08, 0.2)	<0.001	0.16
	T2DM	1023	0.132373	0.941657			
Body of corpus callosum	Non T2DM	30744	-0.010577	0.882668	0.15 (0.09, 0.2)	<0.001	0.17
	T2DM	1023	0.141621	0.945618			
Splenium of corpus callosum	Non T2DM	30744	-0.013656	0.814876	0.07 (0.009, 0.12)	0.02	0.08
	T2DM	1023	0.055546	0.952259			
Fornix	Non T2DM	30744	-0.002883	0.972785	0.1 (0.1, 0.2)	<0.001	0.16
	T2DM	1023	0.158132	0.790076			
Corticospinal tract (R)	Non T2DM	30744	-0.003705	0.976772	0.06 (0.001, 0.12)	0.04	0.06
	T2DM	1023	-0.065183	0.956244			
Corticospinal tract (L)	Non T2DM	30744	-0.005291	0.944221	0.03 (0.03, 0.09)	0.3	0.03
	T2DM	1023	-0.035476	0.977784			
Medial lemniscus (R)	Non T2DM	30744	-0.005511	0.943530	0.02 (0.03, 0.09)	0.3	0.03
	T2DM	1023	0.024341	0.964823			
Medial lemniscus (L)	Non T2DM	30744	-0.006934	0.900660	0.04 (0.01, 0.09)	0.18	0.04
	T2DM	1023	0.033513	0.949495			
Inferior cerebellar (R)	Non T2DM	30744	-0.006924	0.934781	0.06 (0.002, 0.12)	0.04	0.06
	T2DM	1023	0.057846	0.994718			
Inferior cerebellar (L)	Non T2DM	30744	-0.012038	0.857666	0.11 (0.05, 0.17)	<0.001	0.13
	T2DM	1023	0.102156	0.979747			
Superior cerebellar peduncle (R)	Non T2DM	30744	-0.013118	0.844110	0.21 (0.15, 0.27)	<0.001	0.25
	T2DM	1023	0.199189	0.966512			
Superior cerebellar peduncle (L)	Non T2DM	30744	-0.004196	0.889285	0.21 (0.15, 0.27)	<0.001	0.23
	T2DM	1023	0.208803	0.951419			
Cerebral peduncle (R)	Non T2DM	30744	-0.008075	0.931104	0.01 (0.04, 0.07)	0.59	0.01

	T2DM	1023	0.008364	0.954517			
Cerebral peduncle (L)	Non T2DM	30744	-0.008396	0.836658	0.03 (0.02, 0.09)	0.31	0.03
	T2DM	1023	0.022206	0.962617			
Anterior limb of internal capsule (R)	Non T2DM	30744	-0.008823	0.866220	0.05 (0.002, 0.11)	0.03	0.06
	T2DM	1023	0.050778	0.903179			
Anterior limb of internal capsule (L)	Non T2DM	30744	-0.008070	0.850539	0.04 (0.01, 0.10)	0.11	0.05
	T2DM	1023	0.039925	0.850539			
Posterior limb of internal capsule (R)	Non T2DM	30744	-0.007736	0.879087	0.07 (0.02, 0.13)	0.01	0.08
	T2DM	1023	0.070374	0.958745			
Posterior limb of internal capsule (L)	Non T2DM	30744	-0.009929	0.896865	0.09 (0.03, 0.15)	0.002	0.10
	T2DM	1023	0.082606	0.956571			
Retrolenticular part of internal capsule (R)	Non T2DM	30744	-0.010464	0.923660	0.05 (0.009, 0.11)	0.09	0.05
	T2DM	1023	0.043860	1.014059			
Retrolenticular part of internal capsule (L)	Non T2DM	30744	-0.013014	0.846507	0.08 (0.01, 0.14)	0.01	0.09
	T2DM	1023	0.067110	1.017404			
Anterior corona radiata (R)	Non T2DM	30744	-0.013743	0.849947	0.11 (0.05, 0.16)	<0.001	0.13
	T2DM	1023	0.098627	0.912647			
Anterior corona radiata (L)	Non T2DM	30744	-0.013365	0.813163	0.11 (0.05, 0.16)	<0.001	0.13
	T2DM	1023	0.097696	0.927430			
Superior corona radiata (R)	Non T2DM	30744	-0.015028	0.828408	0.10 (0.05, 0.16)	<0.001	0.13
	T2DM	1023	0.094478	0.911509			
Superior corona radiata (L)	Non T2DM	30744	-0.011284	0.855678	0.12 (0.07, 0.18)	<0.001	0.14
	T2DM	1023	0.116498	0.903788			
Posterior corona radiata (R)	Non T2DM	30744	-0.012103	0.875230	0.07 (0.016, 0.13)	0.01	0.08
	T2DM	1023	0.063315	0.945364			
Posterior corona radiata (L)	Non T2DM	30744	-0.010690	0.884201	0.09 (0.03, 0.15)	0.001	0.10
	T2DM	1023	0.083618	0.942719			
Posterior thalamic radiation (R)	Non T2DM	30744	-0.008993	0.875661	0.04 (0.01, 0.09)	0.16	0.04
	T2DM	1023	0.031514	0.921754			
Posterior thalamic radiation (L)	Non T2DM	30744	-0.011944	0.878229	0.03 (0.02, 0.08)	0.29	0.03
	T2DM	1023	0.019197	0.924939			
Sagittal stratum (R)	Non T2DM	30744	-0.011511	0.875434	0.07 (0.01, 0.13)	0.01	0.08
	T2DM	1023	0.060282	0.936762			
Sagittal stratum (L)	Non T2DM	30744	-0.014144	0.847473	0.08 (0.02, 0.14)	0.004	0.09
	T2DM	1023	0.069605	0.910546			
External capsule (R)	Non T2DM	30744	-0.013145	0.848591	0.11 (0.05, 0.17)	<0.001	0.13
	T2DM	1023	0.103757	0.919482			
External capsule (L)	Non T2DM	30744	-0.008395	0.853305	0.11 (0.05, 0.16)	<0.001	0.12
	T2DM	1023	0.102117	0.935666			
Cingulum cingulate gyrus (R)	Non T2DM	30744	-0.007566	0.872258	0.05 (0.005, 0.11)	0.07	0.06
	T2DM	1023	0.046486	0.947063			
Cingulum cingulate gyrus (L)	Non T2DM	30744	-0.002794	0.897824	0.04 (0.01, 0.10)	0.13	0.05
	T2DM	1023	0.043909	0.972382			
Cingulum hippocampus (R)	Non T2DM	30744	-0.004606	0.925845	0.06 (0.001, 0.12)	0.04	0.06
	T2DM	1023	-0.067027	0.963989			
Cingulum hippocampus (L)	Non T2DM	30744	-0.013202	0.835398	0.008 (-0.05, 0.06)	0.7	0.009

	T2DM	1023	-0.005012	0.971394			
Fornix cres+stria terminalis (R)	Non T2DM	30744	-0.011869	0.855022	0.15 (0.09, 0.2)	<0.001	0.17
	T2DM	1023	0.140289	0.937627			
Fornix cres+stria terminalis (L)	Non T2DM	30744	-0.011195	0.878797	0.15 (0.09, 0.2)	<0.001	0.17
	T2DM	1023	0.143742	0.931201			
Superior longitudinal fasciculus (R)	Non T2DM	30744	-0.011695	0.903452	0.05 (0.009, 0.11)	0.09	0.05
	T2DM	1023	0.040456	0.980111			
Superior longitudinal fasciculus (L)	Non T2DM	30744	-0.012387	0.809261	0.06 (0.002, 0.12)	0.03	0.07
	T2DM	1023	0.051710	0.978481			
Superior fronto-occipital fasciculus (R)	Non T2DM	30744	-0.015075	0.836346	0.16 (0.10, 0.21)	<0.001	0.19
	T2DM	1023	0.148578	0.888532			
Superior fronto-occipital fasciculus (L)	Non T2DM	30744	-0.012944	0.888199	0.15 (0.10, 0.21)	<0.001	0.17
	T2DM	1023	0.146217	0.913486			
Uncinate fasciculus (R)	Non T2DM	30744	-0.009583	0.858124	0.09 (0.03, 0.14)	0.002	0.10
	T2DM	1023	0.081419	0.930789			
Uncinate fasciculus (L)	Non T2DM	30744	-0.008948	0.912448	0.04 (0.01, 0.09)	0.14	0.04
	T2DM	1023	0.033251	0.910594			
Tapetum (R)	Non T2DM	30744	-0.007780	0.939976	0.09 (0.03, 0.15)	0.001	0.10
	T2DM	1023	0.090924	0.937740			
Tapetum (L)	Non T2DM	30744	-0.003542	0.755926	0.05 (0.008, 0.10)	0.09	0.06
	T2DM	1023	0.047024	0.939205			

Table S1C. AD and Intergroup Microstructural Differences.

AD WM Tracts and Group		N	Mean	Std. Deviation	Mean Difference (95% CI)	P-value	Effect Size
Middle cerebellar peduncle	Non T2DM	30744	-0.000001	0.872135	0.06 (0.012, 0.11)	0.015	0.06
	T2DM	1023	-0.000001	0.933573			
Pontine crossing tract	Non T2DM	30744	-0.003643	0.923278	0.03 (0.09, 0.016)	0.17	0.04
	T2DM	1023	-0.042206	0.961820			
Genu of corpus callosum	Non T2DM	30744	-0.000660	0.923572	0.06 (0.007, 0.12)	0.02	0.07
	T2DM	1023	0.064089	1.022912			
Body of corpus callosum	Non T2DM	30744	-0.003342	0.875846	0.04 (-0.09, 0.016)	0.16	0.04
	T2DM	1023	0.037294	0.970715			
Splenium of corpus callosum	Non T2DM	30744	-0.011976	0.896345	0.04 (0.008, 0.1)	0.09	0.05
	T2DM	1023	0.035632	0.866163			
Fornix	Non T2DM	30744	0.000987	0.868681	0.12 (0.06, 0.17)	<0.001	0.12
	T2DM	1023	0.122381	0.923362			
Corticospinal tract (R)	Non T2DM	30744	0.000177	0.878636	0.08 (0.02, 0.1)	0.007	0.08
	T2DM	1023	-0.080951	0.970260			
Corticospinal tract (L)	Non T2DM	30744	0.001675	0.872132	0.04 (0.01, 0.1)	0.13	0.04
	T2DM	1023	-0.044352	0.957249			
Medial lemniscus (R)	Non T2DM	30744	0.001575	0.912939	0.04 (0.01, 0.10)	0.16	0.04
	T2DM	1023	-0.042412	0.972131			

Medial lemniscus (L)	Non T2DM	30744	-0.001755	0.861372	0.03 (0.02, 0.09)	0.29	0.04
	T2DM	1023	-0.034810	0.982469			
Inferior cerebellar (R)	Non T2DM	30744	-0.001865	0.868967	0.01 (0.03, 0.06)	0.60	0.01
	T2DM	1023	-0.015933	0.927104			
Inferior cerebellar (L)	Non T2DM	30744	-0.000496	0.911460	0.01 (0.03, 0.07)	0.54	0.02
	T2DM	1023	0.017051	0.941059			
Superior cerebellar peduncle (R)	Non T2DM	30744	0.000941	0.760916	0.02 (0.07, 0.02)	0.28	0.03
	T2DM	1023	0.027590	0.774817			
Superior cerebellar peduncle (L)	Non T2DM	30744	0.006260	0.890016	0.01 (0.03, 0.06)	0.55	0.01
	T2DM	1023	0.022111	0.831469			
Cerebral peduncle (R)	Non T2DM	30744	0.003525	0.928097	0.08 (0.03, 0.14)	0.002	0.09
	T2DM	1023	-0.084260	0.917937			
Cerebral peduncle (L)	Non T2DM	30744	-0.004707	0.828934	0.07 (0.01, 0.12)	0.01	0.08
	T2DM	1023	-0.075310	0.915444			
Anterior limb of internal capsule (R)	Non T2DM	30744	0.018812	0.861586	0.02 (0.03, 0.07)	0.42	0.02
	T2DM	1023	-0.006919	0.903230			
Anterior limb of internal capsule (L)	Non T2DM	30744	0.016098	0.905530	0.02 (0.03, 0.08)	0.44	0.02
	T2DM	1023	-0.008404	0.952195			
Posterior limb of internal capsule (R)	Non T2DM	30744	-0.008404	0.923171	0.13 (0.07, 0.1)	<0.001	0.14
	T2DM	1023	0.127141	1.000958			
Posterior limb of internal capsule (L)	Non T2DM	30744	-0.006181	0.872550	0.15 (0.09, 0.2)	<0.001	0.17
	T2DM	1023	0.149775	0.992516			
Retrolenticular part of internal capsule (R)	Non T2DM	30744	-0.005599	0.883038	0.04 (0.01, 0.1)	0.104	0.05
	T2DM	1023	0.043401	0.944375			
Retrolenticular part of internal capsule (L)	Non T2DM	30744	-0.006309	0.892222	0.08 (0.02, 0.1)	0.008	0.08
	T2DM	1023	0.073864	0.958419			
Anterior corona radiata (R)	Non T2DM	30744	-0.006530	0.903225	0.06 (0.004, 0.12)	0.03	0.07
	T2DM	1023	0.057362	0.942926			
Anterior corona radiata (L)	Non T2DM	30744	-0.009355	0.839222	0.05 (0.009, 0.11)	0.09	0.06
	T2DM	1023	0.041539	0.967223			
Superior corona radiata (R)	Non T2DM	30744	-0.010404	0.844803	0.12 (0.06, 0.1)	<0.001	0.14
	T2DM	1023	0.112374	0.934231			
Superior corona radiata (L)	Non T2DM	30744	-0.011164	0.813793	0.14 (0.08, 0.1)	<0.001	0.17
	T2DM	1023	0.130720	0.919593			
Posterior corona radiata (R)	Non T2DM	30744	-0.011291	0.840908	0.09 (0.04, 0.15)	<0.001	0.11
	T2DM	1023	0.087489	0.902386			
Posterior corona radiata (L)	Non T2DM	30744	-0.000015	0.890276	0.09 (0.03, 0.1)	<0.001	0.10
	T2DM	1023	0.096567	0.918733			
Posterior thalamic radiation (R)	Non T2DM	30744	0.001648	0.887365	0.11 (0.05, 0.1)	<0.001	0.12
	T2DM	1023	-0.108784	0.942285			
Posterior thalamic radiation (L)	Non T2DM	30744	-0.004175	0.850097	0.12 (0.06, 0.18)	<0.001	0.14
	T2DM	1023	-0.127565	0.947306			
Sagittal stratum (R)	Non T2DM	30744	-0.002343	0.849107	0.04 (0.09, 0.01)	0.15	0.04
	T2DM	1023	-0.042466	0.872968			
Sagittal stratum (L)	Non T2DM	30744	-0.008432	0.866533	0.05 (0.005, 0.1)	0.03	0.06

	T2DM	1023	-0.066513	0.841665			
External capsule (R)	Non T2DM	30744	-0.008364	0.872135	0.08 (0.02, 0.1)	0.005	0.09
	T2DM	1023	0.073986	0.933573			
External capsule (L)	Non T2DM	30744	0.003084	0.923278	0.08 (0.02, 0.1)	0.008	0.08
	T2DM	1023	0.084580	0.961820			
Cingulum cingulate gyrus (R)	Non T2DM	30744	0.003921	0.923572	0.04 (0.01, 0.1)	0.17	0.04
	T2DM	1023	-0.040130	1.022912			
Cingulum cingulate gyrus (L)	Non T2DM	30744	-0.001900	0.875846	0.04 (0.01, 0.1)	0.18	0.04
	T2DM	1023	-0.043104	0.970715			
Cingulum hippocampus (R)	Non T2DM	30744	-0.002247	0.896345	0.04 (0.006, 0.1)	0.08	0.05
	T2DM	1023	-0.050638	0.866163			
Cingulum hippocampus (L)	Non T2DM	30744	-0.006947	0.868681	0.01 (0.04, 0.07)	0.56	0.01
	T2DM	1023	-0.024074	0.923362			
Fornix cres+stria terminalis (R)	Non T2DM	30744	-0.003274	0.878636	0.07 (0.01, 0.1)	0.01	0.08
	T2DM	1023	0.074426	0.970260			
Fornix cres+stria terminalis (L)	Non T2DM	30744	-0.006183	0.872132	0.06 (0.004, 0.12)	0.03	0.07
	T2DM	1023	0.058095	0.957249			
Superior longitudinal fasciculus (R)	Non T2DM	30744	-0.006986	0.912939	0.02 (-0.08, 0.03)	0.46	0.02
	T2DM	1023	0.015844	0.972131			
Superior longitudinal fasciculus (L)	Non T2DM	30744	-0.005596	0.861372	0.05 (0.008, 0.11)	0.09	0.06
	T2DM	1023	0.046861	0.982469			
Superior fronto-occipital fasciculus (R)	Non T2DM	30744	-0.009234	0.868967	0.06 (0.01, 0.1)	0.01	0.07
	T2DM	1023	0.060081	0.927104			
Superior fronto-occipital fasciculus (L)	Non T2DM	30744	-0.002873	0.911460	0.08 (0.02, 0.1)	0.007	0.08
	T2DM	1023	0.077354	0.941059			
Uncinate fasciculus (R)	Non T2DM	30744	-0.001580	0.889613	0.03 (0.02, 0.09)	0.25	0.03
	T2DM	1023	0.032790	0.948407			
Uncinate fasciculus (L)	Non T2DM	30744	-0.007549	0.914728	0.003 (-0.05, 0.06)	0.9	0.003
	T2DM	1023	-0.010950	0.934566			
Tapetum (R)	Non T2DM	30744	-0.007161	0.930896	0.10 (0.04, 0.1)	<0.001	0.11
	T2DM	1023	0.097121	0.941111			
Tapetum (L)	Non T2DM	30744	-0.004911	0.945215	0.05 (0.003, 0.11)	0.06	0.05
	T2DM	1023	0.049108	0.914327			

Table S1D. RD and Intergroup Microstructural Differences.

RD WM Tracts and Group		N	Mean	Std. Deviation	Mean Difference (95% CI)	P-value	Effect Size
Middle cerebellar peduncle	Non T2DM	30744	-0.002405	0.845976	0.03 (0.02, 0.09)	0.3	0.03
	T2DM	1023	0.028587	0.949222			
Pontine crossing tract	Non T2DM	30744	-0.013399	0.865917	0.4 (0.03, 0.08)	0.4	0.02
	T2DM	1023	-0.037585	0.903659			
Genu of corpus callosum	Non T2DM	30744	-0.013762	0.905275	0.16 (0.10, 0.22)	<0.001	0.18
	T2DM	1023	0.151666	0.943504			

Body of corpus callosum	Non T2DM	30744	-0.013020	0.888151	0.18 (0.12, 0.2)	<0.001	0.2
	T2DM	1023	0.167642	0.966194			
Splenium of corpus callosum	Non T2DM	30744	-0.014229	0.817146	0.06 (0.01, 0.12)	0.02	0.08
	T2DM	1023	0.055221	0.947744			
Fornix	Non T2DM	30744	-0.005132	0.937023	0.17 (0.12, 0.22)	<0.001	0.18
	T2DM	1023	0.170916	0.781074			
Corticospinal tract (R)	Non T2DM	30744	-0.005926	0.941534	0.03 (0.02, 0.08)	0.2	0.03
	T2DM	1023	-0.038568	0.908045			
Corticospinal tract (L)	Non T2DM	30744	-0.009168	0.889440	0.009 (0.06, 0.04)	0.7	0.01
	T2DM	1023	-0.018883	0.935638			
Medial lemniscus (R)	Non T2DM	30744	-0.009746	0.897430	0.08 (0.02, 0.13)	0.005	0.09
	T2DM	1023	0.071706	0.907780			
Medial lemniscus (L)	Non T2DM	30744	-0.007641	0.872308	0.09 (0.03, 0.15)	<0.001	0.10
	T2DM	1023	0.087385	0.889528			
Inferior cerebellar (R)	Non T2DM	30744	-0.007575	0.874592	0.09 (0.03, 0.14)	<0.001	0.10
	T2DM	1023	0.084823	0.916867			
Inferior cerebellar (L)	Non T2DM	30744	-0.017891	0.938018	0.13 (0.07, 0.18)	<0.001	0.14
	T2DM	1023	0.113452	0.890569			
Superior cerebellar peduncle (R)	Non T2DM	30744	-0.020631	0.920749	0.2 (0.2, 0.3)	<0.001	0.3
	T2DM	1023	0.275214	1.023144			
Superior cerebellar peduncle (L)	Non T2DM	30744	-0.011660	0.874132	0.3 (0.2, 0.3)	<0.001	0.3
	T2DM	1023	0.293889	0.982837			
Cerebral peduncle (R)	Non T2DM	30744	-0.012902	0.821892	0.09 (0.04, 0.15)	<0.001	0.11
	T2DM	1023	0.085740	0.935618			
Cerebral peduncle (L)	Non T2DM	30744	-0.013758	0.724479	0.09 (0.04, 0.15)	<0.001	0.13
	T2DM	1023	0.085820	0.863327			
Anterior limb of internal capsule (R)	Non T2DM	30744	-0.008826	0.801761	0.11 (0.06, 0.15)	<0.001	0.13
	T2DM	1023	0.101212	0.775470			
Anterior limb of internal capsule (L)	Non T2DM	30744	-0.004974	0.833259	0.05 (0.001, 0.11)	0.04	0.06
	T2DM	1023	0.051312	0.872308			
Posterior limb of internal capsule (R)	Non T2DM	30744	-0.002669	0.829215	0.003 (-0.05, 0.05)	0.9	0.004
	T2DM	1023	-0.006060	0.900053			
Posterior limb of internal capsule (L)	Non T2DM	30744	-0.009825	0.891815	0.01 (-0.04, 0.06)	0.6	0.01
	T2DM	1023	-0.020992	0.862397			
Retrolenticular part of internal capsule (R)	Non T2DM	30744	-0.010522	0.906809	0.05 (0.01, 0.11)	0.10	0.05
	T2DM	1023	0.040318	0.986493			
Retrolenticular part of internal capsule (L)	Non T2DM	30744	-0.014973	0.838416	0.06 (0.001, 0.12)	0.04	0.07
	T2DM	1023	0.048049	0.980474			
Anterior corona radiata (R)	Non T2DM	30744	-0.015979	0.831029	0.12 (-0.07, 0.18)	<0.001	0.15
	T2DM	1023	0.111630	0.897157			
Anterior corona radiata (L)	Non T2DM	30744	-0.012253	0.793566	0.13 (-0.07, 0.18)	<0.001	0.16
	T2DM	1023	0.119529	0.895658			
Superior corona radiata (R)	Non T2DM	30744	-0.013764	0.803671	0.07 (0.02, 0.13)	0.006	0.09
	T2DM	1023	0.063695	0.887676			
Superior corona radiata (L)	Non T2DM	30744	-0.009088	0.854033	0.08 (0.02, 0.14)	0.003	0.09

	T2DM	1023	0.074668	0.898453			
Posterior corona radiata (R)	Non T2DM	30744	-0.010442	0.865523	0.04 (0.01, 0.10)	0.10	0.05
	T2DM	1023	0.038683	0.953480			
Posterior corona radiata (L)	Non T2DM	30744	-0.014222	0.868883	0.07 (0.01, 0.13)	0.10	0.08
	T2DM	1023	0.063782	0.942532			
Posterior thalamic radiation (R)	Non T2DM	30744	-0.013086	0.870651	0.11 (0.06, 0.17)	<0.001	0.13
	T2DM	1023	0.103241	0.896766			
Posterior thalamic radiation (L)	Non T2DM	30744	-0.013521	0.887723	0.11 (0.05, 0.16)	<0.001	0.12
	T2DM	1023	0.098984	0.909027			
Sagittal stratum (R)	Non T2DM	30744	-0.014187	0.889908	0.12 (0.06, 0.18)	<0.001	0.13
	T2DM	1023	0.109808	0.916825			
Sagittal stratum (L)	Non T2DM	30744	-0.015120	0.841205	0.14 (0.09, 0.2)	<0.001	0.17
	T2DM	1023	0.133580	0.904474			
External capsule (R)	Non T2DM	30744	-0.013700	0.845976	0.1 (0.06, 0.1)	<0.001	0.14
	T2DM	1023	0.108423	0.893220			
External capsule (L)	Non T2DM	30744	-0.012957	0.873688	0.11 (0.05, 0.16)	<0.001	0.12
	T2DM	1023	0.098110	0.919754			
Cingulum cingulate gyrus (R)	Non T2DM	30744	-0.012227	0.884892	0.10 (0.04, 0.16)	<0.001	0.12
	T2DM	1023	0.097294	0.954022			
Cingulum cingulate gyrus (L)	Non T2DM	30744	-0.002255	0.904744	0.09 (0.03, 0.15)	<0.001	0.10
	T2DM	1023	0.092029	0.960273			
Cingulum hippocampus (R)	Non T2DM	30744	-0.004223	0.907273	0.04 (0.01, 0.1)	0.13	0.05
	T2DM	1023	-0.050656	0.960274			
Cingulum hippocampus (L)	Non T2DM	30744	-0.014456	0.805582	0.02 (0.03, 0.07)	0.4	0.02
	T2DM	1023	0.007244	0.931192			
Fornix cres+stria terminalis (R)	Non T2DM	30744	-0.014156	0.810745	0.17 (0.11, 0.22)	<0.001	0.2
	T2DM	1023	0.157755	0.877533			
Fornix cres+stria terminalis (L)	Non T2DM	30744	-0.012158	0.884928	0.17 (0.11, 0.22)	<0.001	0.19
	T2DM	1023	0.158686	0.878216			
Superior longitudinal fasciculus (R)	Non T2DM	30744	-0.011965	0.884860	0.06 (0.002, 0.12)	0.04	0.07
	T2DM	1023	0.052308	0.981165			
Superior longitudinal fasciculus (L)	Non T2DM	30744	-0.014440	0.811348	0.06 (0.006, 0.12)	0.03	0.08
	T2DM	1023	0.051393	0.954990			
Superior fronto-occipital fasciculus (R)	Non T2DM	30744	-0.016098	0.824481	0.1 (-0.1, 0.2)	<0.001	0.2
	T2DM	1023	0.182561	0.864502			
Superior fronto-occipital fasciculus (L)	Non T2DM	30744	-0.013329	0.889710	0.1 (-0.1, 0.2)	<0.001	0.2
	T2DM	1023	0.177257	0.890326			
Uncinate fasciculus (R)	Non T2DM	30744	-0.011117	0.882107	0.08 (0.02, 0.1)	0.003	0.09
	T2DM	1023	0.072304	0.895438			
Uncinate fasciculus (L)	Non T2DM	30744	-0.008503	0.909535	0.05 (0.003, 0.1)	0.03	0.06
	T2DM	1023	0.050920	0.898001			
Tapetum (R)	Non T2DM	30744	-0.007027	0.926095	0.08 (0.02, 0.1)	0.004	0.09
	T2DM	1023	0.078961	0.943502			
Tapetum (L)	Non T2DM	30744	0.008809	0.934078	0.05 (0.003, 0.1)	0.06	0.05
	T2DM	1023	0.064805	0.956323			

Table S2A. ICVF and Intergroup Microstructural Differences.

ICVF WM Tracts and Group		N	Mean	Std. Deviation	Mean Difference (95% CI)	P-value	Effect Size
Middle cerebellar peduncle	Non T2DM	30744	0.007305	0.935464	0.05 (0.0007, 0.11)	0.05	0.06
	T2DM	1023	-0.052008	0.958151			
Pontine crossing tract	Non T2DM	30744	0.013752	0.947362	0.11 (0.05, 0.17)	<0.001	0.12
	T2DM	1023	-0.102644	0.923836			
Genu of corpus callosum	Non T2DM	30744	0.012168	0.982307	0.09 (0.03, 0.15)	0.002	0.09
	T2DM	1023	-0.083856	1.006732			
Body of corpus callosum	Non T2DM	30744	0.013805	0.934921	0.06 (0.0004, 0.12)	0.04	0.06
	T2DM	1023	-0.050644	1.023081			
Splenium of corpus callosum	Non T2DM	30744	0.013228	0.956534	0.12 (0.06, 0.18)	<0.001	0.12
	T2DM	1023	-0.111102	1.008044			
Fornix	Non T2DM	30744	0.006580	0.939642	0.19 (0.13, 0.26)	<0.001	0.21
	T2DM	1023	-0.192190	0.998659			
Corticospinal tract (R)	Non T2DM	30744	0.003378	0.949858	0.02 (-0.03, 0.08)	0.36	0.02
	T2DM	1023	-0.023140	0.910225			
Corticospinal tract (L)	Non T2DM	30744	0.005813	0.830708	0.02 (-0.02, 0.08)	0.32	0.03
	T2DM	1023	-0.022839	0.915464			
Medial lemniscus (R)	Non T2DM	30744	0.005903	0.808081	0.06 (0.01, 0.12)	0.01	0.08
	T2DM	1023	-0.061863	0.861544			
Medial lemniscus (L)	Non T2DM	30744	0.007553	0.907300	0.04 (-0.002, 0.10)	0.05	0.05
	T2DM	1023	-0.042175	0.822183			
Inferior cerebellar (R)	Non T2DM	30744	0.005544	0.900852	0.05 (-0.006, 0.11)	0.07	0.06
	T2DM	1023	-0.049497	0.976566			
Inferior cerebellar (L)	Non T2DM	30744	0.011320	0.928408	0.08 (0.01, 0.1)	0.01	0.08
	T2DM	1023	-0.068900	0.977558			
Superior cerebellar peduncle (R)	Non T2DM	30744	0.013143	0.951835	0.15 (0.09, 0.22)	<0.001	0.16
	T2DM	1023	-0.145144	0.997603			
Superior cerebellar peduncle (L)	Non T2DM	30744	-0.001466	0.949323	0.16 (0.10, 0.23)	<0.001	0.17
	T2DM	1023	-0.169756	1.040100			
Cerebral peduncle (R)	Non T2DM	30744	0.000089	0.960860	0.06 (0.002, 0.12)	0.06	0.06
	T2DM	1023	0.061296	1.023845			
Cerebral peduncle (L)	Non T2DM	30744	0.009419	0.909809	0.03 (0.02, 0.10)	0.2	0.04
	T2DM	1023	0.048258	1.040465			
Anterior limb of internal capsule (R)	Non T2DM	30744	0.008993	0.913770	0.06 (0.001, 0.12)	0.05	0.06
	T2DM	1023	-0.051018	0.986035			
Anterior limb of internal capsule (L)	Non T2DM	30744	0.012157	0.937554	0.05 (0.01, 0.11)	0.10	0.05
	T2DM	1023	-0.039859	0.997528			
Posterior limb of internal capsule (R)	Non T2DM	30744	0.013524	0.946490	0.08 (0.01, 0.14)	0.01	0.08
	T2DM	1023	-0.067725	1.027955			
Posterior limb of internal capsule (L)	Non T2DM	30744	0.014677	0.945950	0.12 (0.18, 0.05)	<0.001	0.12
	T2DM	1023	-0.106113	1.048330			

Retrolenticular part of internal capsule (R)	Non T2DM	30744	0.014723	0.961711	0.12 (0.06, 0.18)	<0.001	0.12
	T2DM	1023	-0.110239	1.013046			
Retrolenticular part of internal capsule (L)	Non T2DM	30744	0.013687	0.898558	0.11 (0.05, 0.18)	<0.001	0.12
	T2DM	1023	-0.103303	1.021569			
Anterior corona radiata (R)	Non T2DM	30744	0.014989	0.901404	0.11 (0.06, 0.17)	<0.001	0.13
	T2DM	1023	-0.104982	0.951211			
Anterior corona radiata (L)	Non T2DM	30744	0.017582	0.915876	0.12 (0.06, 0.18)	<0.001	0.13
	T2DM	1023	-0.109206	0.958018			
Superior corona radiata (R)	Non T2DM	30744	0.018503	0.906818	0.15 (0.09, 0.21)	<0.001	0.17
	T2DM	1023	-0.139659	0.971252			
Superior corona radiata (L)	Non T2DM	30744	0.013050	0.924112	0.15 (0.09, 0.21)	<0.001	0.16
	T2DM	1023	-0.141316	0.958475			
Posterior corona radiata (R)	Non T2DM	30744	0.013777	0.928688	0.09 (0.02, 0.15)	0.003	0.09
	T2DM	1023	-0.077840	0.992335			
Posterior corona radiata (L)	Non T2DM	30744	0.014517	0.929517	0.09 (0.03, 0.16)	0.001	0.10
	T2DM	1023	-0.084202	0.981826			
Posterior thalamic radiation (R)	Non T2DM	30744	0.013779	0.921806	0.09 (0.03, 0.14)	0.002	0.09
	T2DM	1023	-0.076372	0.947578			
Posterior thalamic radiation (L)	Non T2DM	30744	0.014437	0.942606	0.07 (0.01, 0.13)	0.015	0.07
	T2DM	1023	-0.059944	0.956972			
Sagittal stratum (R)	Non T2DM	30744	0.016588	0.934322	0.09 (0.03, 0.15)	0.001	0.10
	T2DM	1023	-0.080705	0.970561			
Sagittal stratum (L)	Non T2DM	30744	0.015540	0.885872	0.11 (0.05, 0.17)	<0.001	0.13
	T2DM	1023	-0.101366	0.966310			
External capsule (R)	Non T2DM	30744	0.015123	0.879652	0.14 (0.08, 0.19)	<0.001	0.15
	T2DM	1023	-0.125699	0.931929			
External capsule (L)	Non T2DM	30744	0.014958	0.923529	0.14 (0.08, 0.20)	<0.001	0.16
	T2DM	1023	-0.133572	0.943206			
Cingulum cingulate gyrus (R)	Non T2DM	30744	0.016090	0.925023	0.11 (0.05, 0.17)	<0.001	0.12
	T2DM	1023	-0.096621	0.986934			
Cingulum cingulate gyrus (L)	Non T2DM	30744	0.006243	0.918606	0.12 (0.06, 0.18)	<0.001	0.13
	T2DM	1023	-0.118774	0.990442			
Cingulum hippocampus (R)	Non T2DM	30744	0.006594	0.934339	0.009 (0.06, 0.04)	0.7	0.009
	T2DM	1023	-0.002724	0.940100			
Cingulum hippocampus (L)	Non T2DM	30744	0.012025	0.951938	0.01 (-0.04, 0.07)	0.6	0.013
	T2DM	1023	-0.001045	0.955866			
Fornix cres+stria terminalis (R)	Non T2DM	30744	0.010863	0.935298	0.05 (0.006, 0.12)	0.07	0.06
	T2DM	1023	-0.046129	1.009935			
Fornix cres+stria terminalis (L)	Non T2DM	30744	0.013831	0.951016	0.05 (0.01, 0.11)	0.10	0.05
	T2DM	1023	-0.039651	1.024503			
Superior longitudinal fasciculus (R)	Non T2DM	30744	0.015077	0.935487	0.08 (0.01, 0.14)	0.01	0.08
	T2DM	1023	-0.066900	1.037746			
Superior longitudinal fasciculus (L)	Non T2DM	30744	0.015536	0.862444	0.09 (0.03, 0.15)	0.002	0.11
	T2DM	1023	-0.080151	1.007992			
	Non T2DM	30744	0.018558	0.853049	0.18 (0.12, 0.24)	<0.001	0.21

Superior fronto-occipital fasciculus (R)	T2DM	1023	-0.164564	0.920750			
Superior fronto-occipital fasciculus (L)	Non T2DM	30744	0.013831	0.914145	0.18 (0.12, 0.24)	<0.001	0.20
	T2DM	1023	-0.171707	0.905407			
Uncinate fasciculus (R)	Non T2DM	30744	0.014541	0.901027	0.08 (0.03, 0.14)	0.003	0.09
	T2DM	1023	-0.074302	0.937474			
Uncinate fasciculus (L)	Non T2DM	30744	0.008865	0.904342	0.08 (0.02, 0.14)	0.003	0.09
	T2DM	1023	-0.078115	0.922350			
Tapetum (R)	Non T2DM	30744	0.009903	0.911257	0.04 (0.10, -0.01)	0.15	0.04
	T2DM	1023	-0.034038	0.962457			
Tapetum (L)	Non T2DM	30744	-0.002873	0.797617	0.05 (0.001, 0.11)	0.04	0.07
	T2DM	1023	-0.062719	0.931776			

Table S2B. ODI and Intergroup Microstructural Differences.

ODI WM Tracts and Group		N	Mean	Std. Deviation	Mean Difference (95% CI)	P-value	Effect Size
Middle cerebellar peduncle	Non T2DM	30744	-0.005190	0.857172	0.02 (-0.02, 0.08)	0.29	0.03
	T2DM	1023	-0.033455	0.839697			
Pontine crossing tract	Non T2DM	30744	-0.007099	0.941694	0.01 (-0.03, 0.07)	0.49	0.02
	T2DM	1023	0.012605	0.904039			
Genu of corpus callosum	Non T2DM	30744	-0.011982	0.952362	0.08 (0.02, 0.14)	0.005	0.09
	T2DM	1023	0.074936	0.968357			
Body of corpus callosum	Non T2DM	30744	-0.001146	0.907609	0.20 (0.14, 0.26)	<0.001	0.2
	T2DM	1023	0.202081	0.974723			
Splenium of corpus callosum	Non T2DM	30744	-0.009731	0.892025	0.02 (-0.02, 0.08)	0.31	0.03
	T2DM	1023	-0.038655	0.904970			
Fornix	Non T2DM	30744	-0.005118	0.958853	0.11 (0.05, 0.17)	<0.001	0.12
	T2DM	1023	0.110051	0.885119			
Corticospinal tract (R)	Non T2DM	30744	-0.003949	0.968740	0.004 (-0.06, 0.05)	0.86	0.005
	T2DM	1023	0.000980	0.911349			
Corticospinal tract (L)	Non T2DM	30744	-0.010414	0.947906	0.0006 (-0.05, 0.05)	0.98	0.0006
	T2DM	1023	-0.011073	0.938046			
Medial lemniscus (R)	Non T2DM	30744	-0.011443	0.959868	0.13 (0.07, 0.19)	<0.001	0.14
	T2DM	1023	0.124786	0.944640			
Medial lemniscus (L)	Non T2DM	30744	-0.004712	0.845930	0.12 (0.06, 0.18)	<0.001	0.15
	T2DM	1023	0.123324	0.940109			
Inferior cerebellar (R)	Non T2DM	30744	-0.004112	0.880042	0.05 (0.0002, 0.10)	0.05	0.05
	T2DM	1023	0.048501	0.840193			
Inferior cerebellar (L)	Non T2DM	30744	-0.010957	0.893798	0.05 (0.006, 0.11)	0.02	0.06
	T2DM	1023	0.048599	0.847591			
Superior cerebellar peduncle (R)	Non T2DM	30744	-0.013024	0.903371	0.17 (0.11, 0.22)	<0.001	0.18
	T2DM	1023	0.158095	0.901599			
Superior cerebellar peduncle (L)	Non T2DM	30744	-0.012037	0.914330	0.16 (0.10, 0.22)	<0.001	0.17

	T2DM	1023	0.150871	0.959364			
Cerebral peduncle (R)	Non T2DM	30744	-0.011015	0.926919	0.14 (0.08, 0.20)	<0.001	0.15
	T2DM	1023	0.135336	0.934380			
Cerebral peduncle (L)	Non T2DM	30744	-0.008090	0.958322	0.12 (0.06, 0.18)	<0.001	0.13
	T2DM	1023	0.118134	0.951124			
Anterior limb of internal capsule (R)	Non T2DM	30744	-0.010268	0.959925	0.13 (0.07, 0.19)	<0.001	0.14
	T2DM	1023	0.126705	1.000312			
Anterior limb of internal capsule (L)	Non T2DM	30744	0.007647	0.955832	0.12 (0.06, 0.18)	<0.001	0.13
	T2DM	1023	0.133409	0.989242			
Posterior limb of internal capsule (R)	Non T2DM	30744	0.007563	0.962214	0.15 (0.08, 0.21)	<0.001	0.15
	T2DM	1023	-0.143466	1.034879			
Posterior limb of internal capsule (L)	Non T2DM	30744	0.000256	0.926209	0.15 (0.08, 0.21)	<0.001	0.16
	T2DM	1023	-0.150181	1.015262			
Retrolenticular part of internal capsule (R)	Non T2DM	30744	-0.000264	0.917092	0.002 (-0.05, 0.05)	0.94	0.002
	T2DM	1023	-0.002305	0.921240			
Retrolenticular part of internal capsule (L)	Non T2DM	30744	-0.006097	0.973955	0.001 (-0.06, 0.05)	0.97	0.001
	T2DM	1023	-0.007204	0.959180			
Anterior corona radiata (R)	Non T2DM	30744	-0.008931	0.969186	0.05 (-0.003, 0.12)	0.06	0.06
	T2DM	1023	0.049916	1.001484			
Anterior corona radiata (L)	Non T2DM	30744	0.005219	0.942173	0.09 (0.03, 0.15)	0.003	0.09
	T2DM	1023	0.097684	0.993173			
Superior corona radiata (R)	Non T2DM	30744	0.004413	0.939878	0.10 (0.03, 0.16)	0.002	0.10
	T2DM	1023	-0.099003	1.028563			
Superior corona radiata (L)	Non T2DM	30744	0.007727	0.897646	0.11 (0.05, 0.18)	<0.001	0.13
	T2DM	1023	-0.109667	1.027891			
Posterior corona radiata (R)	Non T2DM	30744	0.006224	0.915638	0.07 (0.01, 0.13)	0.011	0.08
	T2DM	1023	-0.071236	0.946985			
Posterior corona radiata (L)	Non T2DM	30744	-0.007952	0.935580	0.04 (0.01, 0.11)	0.12	0.05
	T2DM	1023	-0.056359	0.987689			
Posterior thalamic radiation (R)	Non T2DM	30744	-0.010031	0.939926	0.19 (0.13, 0.25)	<0.001	0.20
	T2DM	1023	0.184935	1.007304			
Posterior thalamic radiation (L)	Non T2DM	30744	-0.003149	0.921666	0.23 (0.17, 0.29)	<0.001	0.25
	T2DM	1023	0.235931	0.954791			
Sagittal stratum (R)	Non T2DM	30744	-0.004774	0.918831	0.10 (0.04, 0.16)	<0.001	0.11
	T2DM	1023	0.098195	0.918600			
Sagittal stratum (L)	Non T2DM	30744	-0.002870	0.925468	0.14 (0.08, 0.20)	<0.001	0.15
	T2DM	1023	0.141023	0.922066			
External capsule (R)	Non T2DM	30744	-0.003427	0.934322	0.0005 (-0.05, 0.05)	0.98	0.0006
	T2DM	1023	-0.003990	0.945501			
External capsule (L)	Non T2DM	30744	-0.010075	0.947605	0.003 (-0.05, 0.06)	0.90	0.003
	T2DM	1023	-0.006377	0.977678			
Cingulum cingulate gyrus (R)	Non T2DM	30744	-0.009250	0.942056	0.10 (0.03, 0.16)	0.002	0.10
	T2DM	1023	0.091791	1.009608			
Cingulum cingulate gyrus (L)	Non T2DM	30744	-0.002937	0.894430	0.08 (0.02, 0.14)	0.008	0.09
	T2DM	1023	0.078417	0.964793			
Cingulum hippocampus (R)	Non T2DM	30744	-0.002711	0.885484	0.03 (0.01, 0.09)	0.16	0.04

	T2DM	1023	0.034950	0.845112			
Cingulum hippocampus (L)	Non T2DM	30744	-0.006460	0.879842	0.05 (0.0008, 0.11)	0.04	0.06
	T2DM	1023	0.049238	0.874294			
Fornix cres+stria terminalis (R)	Non T2DM	30744	-0.007578	0.873578	0.11 (0.05, 0.16)	<0.001	0.12
	T2DM	1023	0.104449	0.893969			
Fornix cres+stria terminalis (L)	Non T2DM	30744	-0.002845	0.968626	0.10 (0.04, 0.15)	<0.001	0.10
	T2DM	1023	0.099052	0.864037			
Superior longitudinal fasciculus (R)	Non T2DM	30744	-0.003203	0.978478	0.04 (0.01, 0.11)	0.12	0.05
	T2DM	1023	0.045510	0.994962			
Superior longitudinal fasciculus (L)	Non T2DM	30744	-0.003849	0.975460	0.001 (-0.06, 0.06)	0.97	0.001
	T2DM	1023	-0.002668	0.986645			
Superior fronto-occipital fasciculus (R)	Non T2DM	30744	-0.004571	0.973629	0.07 (0.016, 0.13)	0.013	0.07
	T2DM	1023	0.072452	0.968336			
Superior fronto-occipital fasciculus (L)	Non T2DM	30744	-0.005291	0.962966	0.07 (0.007, 0.13)	0.028	0.07
	T2DM	1023	0.066096	1.016324			
Uncinate fasciculus (R)	Non T2DM	30744	-0.006810	0.952605	0.03 (0.02, 0.09)	0.24	0.03
	T2DM	1023	0.030030	0.984050			
Uncinate fasciculus (L)	Non T2DM	30744	-0.001043	0.971948	0.05 (0.01, 0.11)	0.10	0.05
	T2DM	1023	0.049051	0.970575			
Tapetum (R)	Non T2DM	30744	0.001961	0.953706	0.003 (-0.05, 0.06)	0.90	0.004
	T2DM	1023	-0.001977	1.019943			
Tapetum (L)	Non T2DM	30744	-0.001174	0.920813	0.01 (0.04, 0.07)	0.52	0.02
	T2DM	1023	-0.020590	0.960777			

Table S2C. IsoVF and Intergroup Microstructural Differences.

IsoVF WM Tracts and Group		N	Mean	Std. Deviation	Mean Difference (95% CI)	P-value	Effect Size
Middle cerebellar peduncle	Non T2DM	30744	0.000395	0.962973	0.02 (0.03, 0.07)	0.46	0.02
	T2DM	1023	0.022047	0.925563			
Pontine crossing tract	Non T2DM	30744	-0.005706	0.842666	0.06 (0.01, 0.12)	0.02	0.08
	T2DM	1023	-0.075122	0.936179			
Genu of corpus callosum	Non T2DM	30744	-0.006912	0.820624	0.13 (0.08, 0.19)	<0.001	0.16
	T2DM	1023	0.132276	0.916119			
Body of corpus callosum	Non T2DM	30744	-0.004719	0.893079	0.15 (0.1, 0.2)	<0.001	0.17
	T2DM	1023	0.154832	0.904973			
Splenium of corpus callosum	Non T2DM	30744	-0.012738	0.805552	0.011 (0.04, 0.07)	0.69	0.01
	T2DM	1023	-0.001215	0.946458			
Fornix	Non T2DM	30744	0.153296	0.986212	0.15 (0.10, 0.20)	<0.001	0.15
	T2DM	1023	-0.002194	0.818421			
Corticospinal tract (R)	Non T2DM	30744	-0.071081	0.987462	0.06 (0.008, 0.12)	0.02	0.06
	T2DM	1023	-0.004541	0.967024			
Corticospinal tract (L)	Non T2DM	30744	-0.046536	0.931960	0.04 (0.10, 0.018)	0.17	0.04
	T2DM	1023	-0.004002	0.965029			
Medial lemniscus (R)	Non T2DM	30744	-0.001475	0.909720	0.002 (-0.05, 0.05)	0.93	0.002

	T2DM	1023	-0.002157	0.907755			
Medial lemniscus (L)	Non T2DM	30744	0.023080	0.955348	0.02 (-0.03, 0.08)	0.37	0.02
	T2DM	1023	-0.003175	0.888243			
Inferior cerebellar (R)	Non T2DM	30744	0.024585	0.923092	0.02 (0.03, 0.09)	0.39	0.02
	T2DM	1023	-0.010869	1.029921			
Inferior cerebellar (L)	Non T2DM	30744	0.052014	0.908929	0.06 (0.001, 0.12)	0.04	0.06
	T2DM	1023	-0.011287	0.980363			
Superior cerebellar peduncle (R)	Non T2DM	30744	0.169801	0.885406	0.18 (0.11, 0.24)	<0.001	0.20
	T2DM	1023	-0.007133	0.995268			
Superior cerebellar peduncle (L)	Non T2DM	30744	0.172560	0.898507	0.17 (0.11, 0.24)	<0.001	0.19
	T2DM	1023	-0.011059	0.964452			
Cerebral peduncle (R)	Non T2DM	30744	0.064267	0.933830	0.07 (0.01, 0.13)	0.01	0.08
	T2DM	1023	-0.004303	0.915960			
Cerebral peduncle (L)	Non T2DM	30744	0.066351	0.872339	0.07 (0.01, 0.12)	0.01	0.08
	T2DM	1023	-0.007063	0.939727			
Anterior limb of internal capsule (R)	Non T2DM	30744	-0.007063	0.913543	0.04 (0.01, 0.10)	0.15	0.04
	T2DM	1023	0.036536	0.947847			
Anterior limb of internal capsule (L)	Non T2DM	30744	-0.000411	0.847361	0.04 (0.02, 0.10)	0.19	0.04
	T2DM	1023	0.040179	0.988671			
Posterior limb of internal capsule (R)	Non T2DM	30744	0.001112	0.895080	0.006 (-0.05, 0.06)	0.81	0.007
	T2DM	1023	0.007967	0.941109			
Posterior limb of internal capsule (L)	Non T2DM	30744	0.000211	0.905044	0.008 (-0.04, 0.06)	0.76	0.009
	T2DM	1023	-0.008758	0.936373			
Retrolenticular part of internal capsule (R)	Non T2DM	30744	0.000216	0.948117	0.02 (-0.03, 0.08)	0.38	0.02
	T2DM	1023	-0.026955	0.979318			
Retrolenticular part of internal capsule (L)	Non T2DM	30744	-0.001685	0.864042	0.003 (-0.05, 0.06)	0.90	0.004
	T2DM	1023	0.002206	0.984961			
Anterior corona radiata (R)	Non T2DM	30744	-0.002656	0.867217	0.03 (0.02, 0.09)	0.28	0.03
	T2DM	1023	0.029989	0.962813			
Anterior corona radiata (L)	Non T2DM	30744	0.000485	0.775599	0.03 (0.02, 0.09)	0.21	0.04
	T2DM	1023	0.037163	0.919515			
Superior corona radiata (R)	Non T2DM	30744	0.000005	0.828937	0.01 (-0.03, 0.07)	0.53	0.02
	T2DM	1023	-0.017272	0.878636			
Superior corona radiata (L)	Non T2DM	30744	-0.000889	0.845752	0.007 (-0.04, 0.06)	0.79	0.008
	T2DM	1023	0.006696	0.917607			
Posterior corona radiata (R)	Non T2DM	30744	-0.000258	0.889272	0.01 (-0.03, 0.07)	0.54	0.02
	T2DM	1023	0.017809	0.926113			
Posterior corona radiata (L)	Non T2DM	30744	0.001612	0.878026	0.02 (-0.03, 0.08)	0.35	0.03
	T2DM	1023	0.029965	0.956673			
Posterior thalamic radiation (R)	Non T2DM	30744	0.001934	0.903640	0.03 (-0.02, 0.09)	0.22	0.04
	T2DM	1023	-0.034446	0.933197			
Posterior thalamic radiation (L)	Non T2DM	30744	0.001111	0.868402	0.02 (-0.03, 0.08)	0.40	0.02
	T2DM	1023	-0.024278	0.957416			
Sagittal stratum (R)	Non T2DM	30744	0.003850	0.914507	0.01 (-0.04, 0.06)	0.67	0.01
	T2DM	1023	0.015848	0.898017			
Sagittal stratum (L)	Non T2DM	30744	-0.006177	0.911352	0.03 (-0.02, 0.09)	0.22	0.04

	T2DM	1023	0.030560	0.955080			
External capsule (R)	Non T2DM	30744	-0.006278	0.927114	0.05 (0.01, 0.11)	0.10	0.05
	T2DM	1023	0.045566	0.994139			
External capsule (L)	Non T2DM	30744	0.004396	0.884328	0.05 (0.01, 0.11)	0.10	0.05
	T2DM	1023	0.056082	0.987675			
Cingulum cingulate gyrus (R)	Non T2DM	30744	0.007014	0.897091	0.02 (0.03, 0.08)	0.36	0.03
	T2DM	1023	-0.020400	0.946805			
Cingulum cingulate gyrus (L)	Non T2DM	30744	-0.004294	0.953645	0.04 (0.01, 0.10)	0.14	0.04
	T2DM	1023	-0.049456	0.978928			
Cingulum hippocampus (R)	Non T2DM	30744	-0.008083	0.931541	0.02 (-0.03, 0.08)	0.38	0.02
	T2DM	1023	0.018931	0.967138			
Cingulum hippocampus (L)	Non T2DM	30744	-0.008989	0.862395	0.11 (0.05, 0.16)	<0.001	0.12
	T2DM	1023	0.101430	0.944290			
Fornix cres+stria terminalis (R)	Non T2DM	30744	-0.008366	0.862746	0.13 (0.07, 0.19)	<0.001	0.15
	T2DM	1023	0.126734	0.951706			
Fornix cres+stria terminalis (L)	Non T2DM	30744	-0.001220	0.833280	0.13 (0.07, 0.18)	<0.001	0.15
	T2DM	1023	0.129595	0.933310			
Superior longitudinal fasciculus (R)	Non T2DM	30744	0.000831	0.922646	0.004 (-0.05, 0.06)	0.86	0.005
	T2DM	1023	-0.003976	0.904307			
Superior longitudinal fasciculus (L)	Non T2DM	30744	-0.002597	0.883867	0.01 (-0.04, 0.07)	0.61	0.01
	T2DM	1023	-0.018648	1.003450			
Superior fronto-occipital fasciculus (R)	Non T2DM	30744	-0.004833	0.901788	0.06 (0.006, 0.12)	0.03	0.07
	T2DM	1023	0.061579	0.962467			
Superior fronto-occipital fasciculus (L)	Non T2DM	30744	-0.003185	0.958573	0.06 (0.002, 0.12)	0.04	0.06
	T2DM	1023	0.062018	0.995493			
Uncinate fasciculus (R)	Non T2DM	30744	-0.003881	0.962372	0.05 (0.002, 0.11)	0.06	0.05
	T2DM	1023	0.053758	0.961407			
Uncinate fasciculus (L)	Non T2DM	30744	-0.005729	0.944641	0.01 (-0.04, 0.07)	0.65	0.01
	T2DM	1023	0.008154	0.985841			
Tapetum (R)	Non T2DM	30744	-0.002269	0.955953	0.10 (0.04, 0.16)	<0.001	0.10
	T2DM	1023	0.099335	0.966802			
Tapetum (L)	Non T2DM	30744	-0.002269	0.955953	0.01 (-0.04, 0.07)	0.66	0.01
	T2DM	1023	0.011437	0.989257			

Table S3. Associations between disease duration/HbA1c and White Matter changes in T2DM participants. NS= not significant.

WM Tracts and Fibres			FA		MD		AD		RD		ICVF		ODI		IsoVF	
			Disease duration	Hb1Ac	Disease duration	Hb1Ac	Disease duration	Hb1Ac	Disease duration	Hb1Ac	Disease duration	Hb1Ac	Disease duration	Hb1Ac	Disease duration	Hb1Ac
	Middle cerebellar peduncle	Correlation (r)	NS	NS	NS	NS	0.07	0.01	NS	NS	NS	NS	NS	NS	NS	NS
		P-value					0.02	0.001								
	Pontine crossing tract	Correlation (r)	NS	NS	NS	NS	NS	NS	NS	NS	-0.06	-0.01	NS	NS	NS	NS
		P-value									0.04	0.001				

	Genu of corpus callosum	Correlation (r)	-0.03	-0.02	0.11	0.02	0.01	0.006	0.08	0.02	-0.08	-0.01	-0.01	0.01	0.09	0.01
		P-value	0.22	<0.001	<0.001	<0.001	<0.001	0.2	0.005	<0.001	0.005	0.002	0.59	0.06	0.002	0.002
	Body of corpus callosum	Correlation (r)	-0.06	-0.02	0.09	0.01	NS	NS	0.08	0.02	-0.07	-0.01	0.02	0.02	0.05	0.01
		P-value	0.02	<0.001	0.002	0.003			0.005	<0.001	0.01	0.007	0.46	<0.001	0.06	0.05
	Splenium of the corpus callosum	Correlation (r)	-0.06	-0.01	0.11	0.019	NS	NS	0.10	0.01	-0.1	-0.02	NS	NS	NS	NS
		P-value	0.03	<0.001	<0.001	<0.001			<0.001	<0.001	<0.001	<0.001				
	Fornix	Correlation (r)	-0.08	-0.02	0.04	0.01	0.05	0.007	0.04	0.01	-0.05	-0.02	0.001	0.01	0.05	0.01
		P-value	0.007	<0.001	0.13	0.01	0.07	0.18	0.2	<0.001	0.06	<0.001	0.97	0.003	0.11	0.01
	Corticospinal tract (R)	Correlation (r)	NS	NS	NS	NS	0.04	-0.004	NS	NS	NS	NS	NS	NS	0.01	-0.002
		P-value					0.1	0.4							0.6	0.63
	Corticospinal tract (L)	Correlation (r)	NS	NS	NS	NS	NS	NS	NS	NS	NS	NS	NS	NS	NS	NS
		P-value														
	Medial lemniscus (R)	Correlation (r)	-0.07	-0.02	NS	NS	NS	NS	0.07	0.02	-0.08	-0.02	0.04	0.008	NS	NS
		P-value	0.02	<0.001					0.01	<0.001	0.006	<0.001	0.17	0.16		
	Medial lemniscus (L)	Correlation (r)	-0.08	-0.02	NS	NS	NS	NS	0.07	0.02	NS	NS	0.03	0.02	NS	NS
		P-value	0.009	<0.001					0.02	<0.001			0.24	<0.001		
	Inferior cerebellar peduncle (R)	Correlation (r)	-0.08	-0.01	0.07	0.01	NS	NS	0.07	0.02	NS	NS	NS	NS	NS	NS
		P-value	0.009	0.003	0.01	0.003			0.01	<0.001						
	Inferior cerebellar peduncle (L)	Correlation (r)	-0.05	-0.02	0.07	0.03	NS	NS	0.07	0.03	-0.06	-0.02	0.01	0.01	0.01	0.01
		P-value	0.08	<0.001	0.01	<0.001			0.01	<0.001	0.03	<0.001	0.57	0.07	0.62	0.005
	Superior cerebellar peduncle (R)	Correlation (r)	-0.05	-0.04	0.09	0.03	NS	NS	0.09	0.04	-0.02	-0.02	0.03	0.02	0.09	0.03
		P-value	0.09	<0.001	0.002	<0.001			0.002	<0.001	0.46	<0.001	0.21	<0.001	0.003	<0.001
	Superior cerebellar peduncle (L)	Correlation (r)	-0.07	-0.03	0.06	0.01	NS	NS	0.07	0.03	-0.03	-0.01	0.03	0.02	0.05	0.01
		P-value	0.02	<0.001	0.05	0.003			0.02	<0.001	0.24	0.02	0.2	<0.001	0.10	0.002
	Cerebral peduncle (R)	Correlation (r)	-0.06	-0.01	0.05	-0.003	-0.01	-0.01	0.09	0.01	NS	NS	0.04	0.02	0.02	0.001
		P-value	0.03	0.003	0.08	0.51	0.5	0.001	0.002	0.02			0.16	<0.001	0.42	0.79
	Cerebral peduncle (L)	Correlation (r)	-0.09	-0.01	0.02	0.004	-0.02	-0.01	0.09	0.01	NS	NS	0.04	0.01	0.005	0.008
		P-value	<0.001	<0.001	0.36	0.45	0.4	0.06	0.003	0.004			0.12	0.001	0.85	0.12
	Anterior limb of internal capsule (R)	Correlation (r)	-0.080	-0.01	0.03	0.007	NS	NS	0.08	0.01	NS	NS	-0.003	0.01	NS	NS
		P-value	0.005	0.01	0.20	0.20			0.009	0.006			0.9	0.02		
	Anterior limb of internal capsule (L)	Correlation (r)	-0.03	-0.005	NS	NS	NS	NS	0.04	-0.002	NS	NS	0.02	0.01	NS	NS
		P-value	0.3	0.35					0.19	0.7			0.47	0.01		
	Posterior limb of internal capsule (R)	Correlation (r)	NS	NS	0.08	0.006	0.03	0.01	NS	NS	-0.09	-0.01	-0.01	-0.02	NS	NS
		P-value			0.005	0.24	0.2	0.002			0.003	0.01	0.5	<0.001		
	Posterior limb of internal capsule (L)	Correlation (r)	-0.05	0.007	NS	NS	0.02	0.02	NS	NS	-0.09	-0.02	-0.01	-0.02	NS	NS
		P-value	0.07	0.2			0.5	<0.001			0.002	<0.001	0.6	<0.001		
	Retrolenticular part of internal capsule R	Correlation (r)	NS	NS	NS	NS	NS	NS	NS	NS	-0.07	-0.02	NS	NS	NS	NS
		P-value									0.01	<0.001				
	Retrolenticular part of internal capsule (L)	Correlation (r)	NS	NS	0.02	0.01	-0.02	0.008	0.04	0.01	-0.08	-0.02	NS	NS	NS	NS
		P-value			0.5	0.004	0.4	0.18	0.18	0.001	0.007	<0.001				
	Anterior corona radiata (R)	Correlation	-0.05	-0.02	0.11	0.02	0.08	0.01	0.11	0.02	-0.12	-0.02	NS	NS	NS	NS
		P-value	0.10	<0.001	<0.001	<0.001	0.005	0.01	<0.001	<0.001	<0.001	<0.001				
	Anterior corona radiata (L)	Correlation (r)	-0.09	-0.01	0.10	0.02	NS	NS	0.10	0.02	-0.10	-0.02	0.007	-0.001	NS	NS
		P-value	0.003	0.001	<0.001	<0.001			<0.001	<0.001	<0.001	<0.001	0.81	0.8		
	Superior corona radiata (R)	Correlation (r)	NS	NS	0.09	0.02	0.04	0.02	0.10	0.01	-0.10	-0.02	-0.001	-0.01	NS	NS
		P-value			0.001	<0.001	0.12	<0.001	<0.001	0.003	<0.001	<0.001	0.9	0.009		

	Superior corona radiata (L)	Correlation (r)	NS	NS	0.08	0.02	0.04	0.02	0.08	0.01	-0.09	-0.02	-0.004	-0.02	NS	NS
		P-value			0.008	<0.001	0.18	<0.001	0.006	0.003	0.001	<0.001	0.8	<0.001		
	Posterior corona radiata (R)	Correlation (r)	NS	NS	0.09	0.01	0.07	0.02	NS	NS	-0.09	-0.01	-0.02	-0.01	NS	NS
		P-value			0.004	<0.001	0.02	<0.001			0.002	<0.001	0.4	0.005		
	Posterior corona radiata (L)	Correlation (r)	NS	NS	0.06	0.01	0.05	0.01	0.06	0.01	-0.08	-0.01	NS	NS	NS	NS
		P-value			0.03	<0.001	0.06	0.07	0.03	0.001	0.005	0.013				
	Posterior thalamic radiation (R)	Correlation (r)	-0.08	-0.02	NS	NS	0.01	-0.008	0.09	0.02	-0.08	-0.01	-0.02	0.02	NS	NS
		P-value	0.005	<0.001			0.58	0.15	0.002	<0.001	0.005	0.002	0.45	<0.001		
	Posterior thalamic radiation (L)	Correlation (r)	-0.08	-0.03	NS	NS	-0.03	-0.01	0.07	0.02	-0.06	-0.02	0.08	0.03	NS	NS
		P-value	0.005	<0.001			0.28	0.07	0.01	<0.001	0.04	<0.001	0.01	<0.001		
	Sagittal stratum (R)	Correlation (r)	-0.08	-0.02	0.07	0.01	NS	NS	0.08	0.02	-0.07	-0.02	0.04	0.01	NS	NS
		P-value	0.01	<0.001	0.01	0.006			0.004	<0.001	0.01	<0.001	0.14	0.01		
	Sagittal stratum (L)	Correlation (r)	-0.05	-0.03	0.06	0.01	NS	NS	0.07	0.02	-0.07	-0.02	0.05	0.02	NS	NS
		P-value	0.08	<0.001	0.03	0.001			0.02	<0.001	0.01	<0.001	0.08	<0.001		
	External capsule (R)	Correlation (r)	-0.07	-0.01	0.07	0.01	0.06	-0.002	0.08	0.02	-0.14	-0.03	NS	NS	NS	NS
		P-value	0.01	<0.001	0.01	0.002	0.04	0.70	0.008	<0.001	<0.001	<0.001				
	External capsule (L)	Correlation (r)	-0.11	-0.01	0.08	0.01	0.03	-0.008	0.10	0.01	-0.14	-0.02	NS	NS	NS	NS
		P-value	<0.001	0.02	0.007	0.007	0.22	0.17	0.001	0.004	<0.001	<0.001				
	Cingulum cingulate gyrus R	Correlation (r)	-0.08	-0.01	NS	NS	NS	NS	0.09	0.01	-0.12	-0.02	0.04	0.007	NS	NS
		P-value	0.008	0.003					0.003	0.005	<0.001	<0.001	0.11	0.17		
	Cingulum cingulate gyrus (L)	Correlation (r)	-0.08	-0.01	NS	NS	NS	NS	0.08	0.01	-0.13	-0.01	0.03	0.01	NS	NS
		P-value	0.008	<0.001					0.004	0.02	<0.001	0.001	0.25	0.01		
	Cingulum hippocampus (R)	Correlation (r)	NS	NS	0.06	-0.002	NS	NS	NS	NS	NS	NS	NS	NS	NS	NS
		P-value			0.05	0.62										
	Cingulum hippocampus (L)	Correlation (r)	NS	NS	NS	NS	NS	NS	NS	NS	NS	NS	-0.03	0.01	0.02	0.02
		P-value											0.26	0.009	0.35	<0.001
	Fornix cresstria terminalis (R)	Correlation (r)	-0.08	-0.03	0.09	0.03	0.05	0.009	0.10	0.03	NS	NS	0.02	0.03	0.07	0.02
		P-value	0.005	<0.001	<0.001	<0.001	0.07	0.13	0.001	<0.001			0.4	<0.001	0.01	<0.001
	Fornix cresstria terminalis (L)	Correlation (r)	-0.06	-0.02	0.07	0.02	0.03	0.01	0.07	0.03	NS	NS	0.01	0.01	0.04	0.02
		P-value	0.03	<0.001	0.02	<0.001	0.27	0.04	0.01	<0.001			0.61	0.02	0.14	<0.001
	Superior longitudinal fasciculus (R)	Correlation (r)	-0.11	-0.02	NS	NS	NS	NS	0.09	0.02	-0.10	-0.02	NS	NS	NS	NS
		P-value	<0.001	<0.001					0.002	<0.001	<0.001	<0.001				
	Superior longitudinal fasciculus (L)	Correlation (r)	-0.08	-0.02	0.05	0.02	NS	NS	0.06	0.02	-0.09	-0.02	NS	NS	NS	NS
		P-value	0.01	<0.001	0.06	<0.001			0.02	<0.001	0.001	<0.001				
	Superior fronto-occipital fasciculus (R)	Correlation (r)	-0.09	-0.03	0.10	0.03	0.08	0.02	0.10	0.04	-0.13	-0.03	0.06	0.001	0.01	0.01
		P-value	<0.001	<0.001	<0.001	<0.001	0.009	<0.001	<0.001	<0.001	<0.001	<0.001	0.03	0.79	0.57	0.03
	Superior fronto-occipital fasciculus (L)	Correlation (r)	-0.07	-0.02	0.07	0.02	0.04	0.01	0.07	0.02	-0.11	-0.03	-0.01	0.007	0.02	0.005
		P-value	0.01	<0.001	0.02	<0.001	0.16	0.01	0.01	<0.001	<0.001	<0.001	0.53	0.17	0.48	0.35
	Uncinate fasciculus (R)	Correlation (r)	-0.04	-0.007	0.06	0.02	NS	NS	0.06	0.01	-0.11	-0.02	NS	NS	NS	NS
		P-value	0.14	0.23	0.04	<0.001			0.03	0.01	<0.001	<0.001				
	Uncinate fasciculus (L)	Correlation (r)	-0.03	-0.01	NS	NS	NS	NS	0.05	0.01	-0.08	-0.01	NS	NS	NS	NS
		P-value	0.24	0.02					0.06	0.03	0.005	0.002				
	Tapetum (R)	Correlation (r)	-0.10	-0.01	0.12	0.01	0.09	0.01	0.12	0.01	NS	NS	NS	NS	NS	NS
		P-value	<0.001	0.004	<0.001	<0.001	0.002	0.005	<0.001	0.001						
	Tapetum (L)	Correlation (r)	NS	NS	NS	NS	NS	NS	NS	NS	-0.10	-0.005	NS	NS	0.08	0.006
		P-value									<0.001	0.34			0.008	0.29
